# Selective Gene Loss of Visual and Olfactory Guanylyl Cyclase Genes Following the Two Rounds of Vertebrate-Specific Whole-Genome Duplications

**DOI:** 10.1093/gbe/evaa192

**Published:** 2020-09-11

**Authors:** Matthias Gesemann, Stephan C F Neuhauss

**Affiliations:** Institute of Molecular Life Sciences, University of Zurich, Switzerland

**Keywords:** guanylyl cyclase, vertebrates, vision, olfaction, gene loss, whole-genome duplication

## Abstract

Photoreceptors convey visual information and come in two flavors; dim-light and bright-light dedicated rod and cones. Both cell types feature highly specialized phototransduction cascades that convert photonic energy into intracellular signals. Although a substantial amount of phototransduction gene ohnologs are expressed either in rods or cones, visual guanylyl cyclases (GCs) involved in the calcium (Ca^2+^) dependent feedback regulation of phototransduction are neither rod nor cone specific. The co-existence of visual GCs in both photoreceptor types suggests that specialization of these ohnologs occurred despite their overlapping expression.

Here, we analyze gene retention and inactivation patterns of vertebrate visual and closely related olfactory GCs following two rounds (2R) of vertebrate-specific whole-genome duplication events (2R WGD). Although eutherians generally use two visual and one olfactory GC, independent inactivation occurred in some lineages. Sauropsids (birds, lizards, snakes, turtles, and crocodiles) generally have only one visual GC (GC-E). Additionally, turtles (testodes) also lost the olfactory GC (GC-D). Pseudogenization in mammals occurred in specific species/families likely according to functional needs (i.e., many species with reduced vision only have GC-E). Likewise, some species not relying on scent marks lack GC-D, the olfactory GC enzyme. Interestingly, in the case of fish, no species can be found with fewer than three (two visual and one olfactory) genes and the teleost-specific 3R WGD can increase this number to up to five. This suggests that vision in fish now requires at least two visual GCs.

**Significance**

Whole-genome duplications (WGDs) are drastic genome reshaping events, which occurred twice at the base of the vertebrate linage and up to two additional times in teleost fish. Such duplication events enable paralogous genes to adopt novel functions and/or share existing ones. Gene loss triggered by regressive evolution might present an additional way to shape gene families. In the case of guanylyl cyclases, WGDs established two visual and one olfactory cyclase at the base of the jawed vertebrates linage. In this report, we trace the presence of these genes following the two rounds (2R) of vertebrate WGD and correlate gene loss and duplication to adaptation in life style. Our results suggest that the functional range of these enzymes can be expanded, subdivided or shared, dependent on species-specific needs.

## Introduction

Developing a sensory system to detect light, picture the surrounding environment, and sense motion within its vicinity is one of the key inventions of evolution. Over time, specialized cells called photoreceptors, being entirely committed towards the detection of photons have evolved. In the vertebrate retina, not only one but two different types of photoreceptors are present (Lamb 2013). While rod photoreceptors are specialized for dim light vision, cone photoreceptors have adapted to transmit color information during daylight. In general, phototransduction (the signaling cascade transmitting photonic energy into changes in neurotransmitter release) in cones and rods follows a similar path (reviewed in Lamb 2013). However, although both types of photoreceptors rely on nearly identical mechanisms, the different cell types use slightly modified, but related, sets of genes and proteins to achieve phototransduction. These genes are usually ohnologs arising from two rounds of whole-genome duplications (2R WGD) that occurred at the base of the vertebrate linage. WGDs and the generation of ohnologs are thought to be one of the main driving forces of evolutionary adaptation (Ohno 1970). Duplicated genes are free to distribute certain function of the ancestral gene (subfunctionalization), adopt new functions (neofunctionalization) or loose the function of one ohnolog (nonfunctionalization) (Glasauer and Neuhauss 2014). Several previous studies have already demonstrated that 2R WGD have extensively expanded the number of genes involved in sensory systems in general (Sato et al. 2009) and phototransduction in particular (Nordström et al. 2004; Larhammar et al. 2009; Lagman et al. 2012). Moreover, the pattern of duplication, consolidation and nonfunctionalization of genes involved in phototransduction predating the completion of 2R WGDs have been studied in great detail (Lamb 2013; Lamb et al. 2016; Lamb and Kraft 2016; Lamb et al. 2018; Lamb and Hunt 2018). Nevertheless, the fate of phototransduction gene ohnologs in different vertebrate lineages following the 2R WGD has not been systematically investigated. In this study, we now analyzed gene preservation, gene loss, and gene duplication of guanylyl cyclases (GCs) following 2R WGD.

Many duplicated genes involved in the activation (e.g., opsins, transducins, phosphodiesterases, or cyclic nucleotide gated channels) or shut-off (e.g., GRKs, arrestins and RGS9) of phototransduction, are expressed specifically in either rods or cones. In contrast, visual GC that are involved in the Ca^2+^ feedback regulation are usually neither rod nor cone specific (Lamb 2013).

Although GC-E is present in rods and cones (Dizhoor et al. 1994), GC-F is predominantly expressed in rods (Yang et al. 1995; Yang and Garbers 1997). Expression levels of the visual GC-E seem to be several fold higher and more widespread when compared with the visual GC-F (Helten et al. 2007). The disruption of the GC-E encoding gene *Gucy2e* in mice resulted in severe cone dystrophy leaving rod morphology largely unchanged (Yang et al. 1999). This finding suggests that GC-F cannot compensate for GC-E in cones but may do so in rods. Double knockouts of *Gucy2e* and *Gucy2f* resulted in a complete absence of light induced electrical responses and the subsequent degeneration of both rods and cones (Baehr et al. 2007). However, human patients with *GUCY2F* mutations that affect vision have so far not been described, suggesting that GC-F function in the primate visual system maybe redundant. In contrast to this, mutations in *GUCY2E* (originally called *GUCY2D*) result in multiple severe phenotypes falling into a different category of disorders known as Leber congenital amaurosis type 1 (LCA1), cone-rod dystrophies or retinitis pigmentosa, which are all characterized by early onset photoreceptor degeneration (Perrault et al. 1996; Kuhn 2016; Sharon et al. 2018). Around 10% of all LCA1 cases can be attributed to mutations in *GUCY2E(D)* (Jacobson et al. 2013) making it the gene most frequently affected in this childhood disease.

Phylogenetically, the two retinal GCs (GC-E, GC-F) and the olfactory GC (GC-D) are closely related. Remarkably, the visual GCs are more closely related to the olfactory GC-D than to each other. However, although retinal GCs are activated by GC activating proteins (GCAPs), the olfactory GC-D acts independently from these activators (Kuhn 2016).

Nevertheless, functionally there might also be some relation between these two groups of cyclases as GC-D expression in the retina of some basal fish species has recently been described (Lamb and Hunt 2018). In general, GC-D expression is restricted to some special cells in the olfactory epithelium (OE), where their signaling represents an alternative to the cAMP signaling. OE cells expressing GC-D lack the regular signaling components used in the cAMP pathway. Instead they use CNGA3 to regulate intracellular calcium levels, similar to retinal neurons (Fülle et al. 1995; Juilfs et al. 1997). GC-D positive epithelial cells project axons to the neckless glomeruli at the caudal end of the olfactory bulb (Juilfs et al. 1997). These neurons participate in sensing the peptide hormones uroguanylin and guanylin present in urine and feces (Leinders-Zufall et al. 2007). Furthermore, GC-D positive cells play a role in detecting different concentrations of CO_2_ (Hu et al. 2007). In contrast to *Gucy2e* deficient mice, mice lacking *Gucy2d* show only subtle defects such as the lack of odor dependent alteration in food related social learning and a reduced capability in CO_2_ detection (Hu et al. 2007; Arakawa et al. 2013).

In the present study, we have analyzed the evolution of visual and olfactory GCs in >300 different vertebrate species following 2R WGD. Although we see a number of gene inactivation events occurring independently in different vertebrates lineages, a remarkable number of species retain two visual GCs. This suggests that alterations in gene content might be attributed to adaptation to life style, social behavior or food acquiring preferences; whereas in some cases, the absence is best explained as a consequence of gene inactivation occurring in “bottleneck” species subsequently influencing the entire downstream clade.

## Materials and Methods

### Annotation of GC Sequences

As gene and cDNA sequence predictions produced by automated processes have been shown to contain numerous errors, GC cDNA sequences used in this study were manually annotated. Sequences were identified and annotated using combined information from expressed sequence tags and genome databases (GeneBank, http://www.ncbi.nlm.nih.gov, last accessed July 8, 2020; Ensembl, http://www.ensembl.org/index.html, last accessed July 8, 2020). Human and mouse sequences were used as initial query (for more details on sequence annotation, see [Gesemann et al. 2010]). Putative frame shift and nonsense mutations within the manually annotated sequences were verified using whole-genome shotgun (WGS) sequences and/or additional sequence information from expressed sequence tags. Moreover, only sequences with more than one inactivating mutation or with similar corresponding mutations in closely related species were considered functionally relevant. Genomes with poor sequence quality (multiple Ns in the extracted sequence or large sequence parts missing) were excluded from the analysis. Exon sizes as well as putative cDNA sequences of GCs from related species were used as additional reference. Intron/Exon boundaries were determined using a combination of the genescan webpage (http://hollywood.mit.edu/GENSCAN.html) and manual inspection analyzing major as well as minor spice site consensus sequences (Patel and Steitz 2003). A complete list of all the assembled sequences, including intron and exons sizes and putative inactivating mutations, is given in the Supplementary Material online.

### Phylogenetic Tree Analysis

Phylogenetic analysis was performed on the Phylogeny.fr plat-form (http://www.phylogeny.fr/, last accessed July 8, 2020) (Dereeper et al. 2008). For protein sequence based phylogenies GC cDNA sequences covering the first three exons were translated into amino acid sequences and subsequently aligned using MUSCLE v3.7 (Edgar 2004) configured for highest accuracy (MUSCLE with default set-tings). Length of input sequences varied between 402 and 515 amino acids. After alignment, ambiguous regions (i.e., containing gaps and/or being poorly aligned) were removed with Gblocks v0.91b (Castresana 2000) using the following parameters: The minimal length of a block after gap clearing was set to 5 and no gap positions in the final alignment were allowed. Alignments with continuous nonconserved positions >8 were rejected and at least 55% of the sequences had to be present at gap flanking positions. Following curation 335 amino acids were used for further analysis. Phylogenetic trees reconstructions were done using the maximum-likelihood method implemented in the PhyML program v3.0 (Guindon and Gascuel 2003). The gamma shape parameter was estimated directly from the data. Branch reliability was assessed by the approximate like-lihood-ratio test (aLRT, SH-like) (Anisimova and Gascuel 2006). Graphical representations of the phylogenetic trees were obtained using TreeDyn v198.3 and edited in Coral draw (CoralCorporation). For DNA sequence based phylogenies, GC cDNA sequences covering the first three exons were aligned using MUSCLE v3.7 (Edgar 2004) configured for highest accuracy (MUSCLE with default set-tings). Fasta files of the input sequences are given in the Supplementary Material online.

### Synteny Analysis

An initial rough synteny analysis was done using the synteny database (http://syntenydb.uoregon.edu/synteny_db/, last accessed July 8, 2020) (Catchen et al. 2009). Synteny hits in the output files were further subjected to a microsynteny analyses, were paralogous/orthologous genes, were used as initial queries for a tBLASTx search against the NCBI database (ncbi nr/nt data-base). The hit with the highest conservation (length and identity) was used in a reciprocal tBLASTx search against the corresponding database and only genes identifying the initially used query are counted as positive matches.

## Results and Discussion

We set out to investigate the preservation, inactivation and potential functional specification of retinal and olfactory GC’s across different vertebrate species. Thus, we assembled and analyzed sequences of >1,000 different genes, covering >300 vertebrate species including mammals, birds, reptiles, amphibians, and fish.

**Fig. 1.**
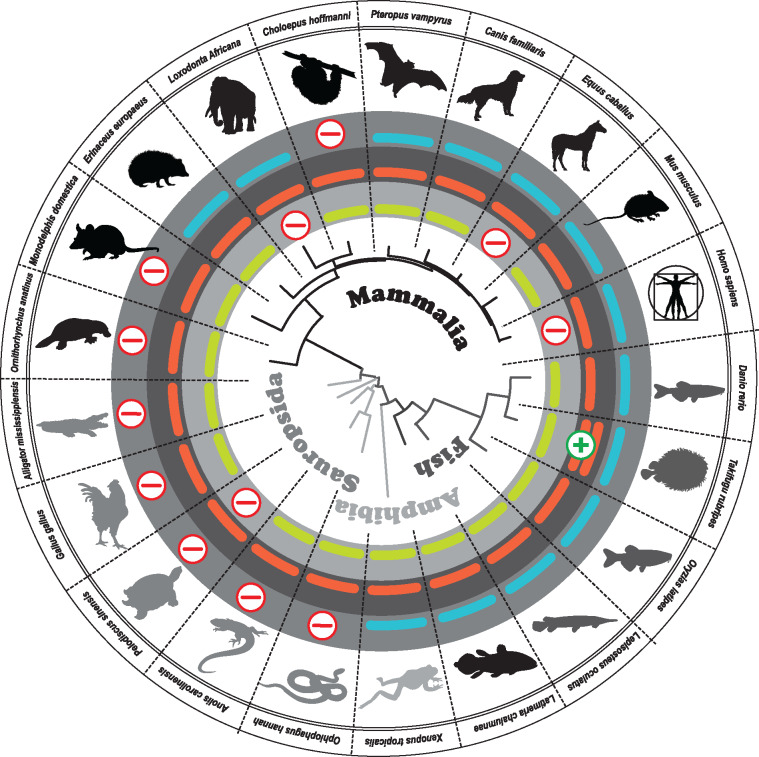
Vertebrates show frequent inactivation of guanylyl cyclase genes. In the center, the GC-E phylogeny of major representatives of the main vertebrate orders is given. The name of each order is shown in different gray intensities and the corresponding members of the orders are colored accordingly. Pictograms for each chosen representative are shown and scientific names are given. The presence of an intact olfactory guanylyl cyclase GC-D is depicted by a light green bar, intact visual GC-E is shown as a red bar and GC-F genes without inactivating mutation are illustrated in blue. Absent or inactivated genes are highlighted by an encircled red minus sign and duplicated genes are given by an encircled green plus sign. Note that in sauropsids a clear pattern of inactivation is already apparent in this survey analysis whereas for neither fish nor mammals an obvious pattern is discernible.

In general, retinal and olfactory GC (*GUCY2*) genes consist of 16 exons, encoding roughly 1,100 amino acids. Although the C-terminal part of the proteins shares a high degree of homology, the N-terminal portion is more variable (supplement S1, Supplementary Material online). As phylogenetic analysis of closely related sequences often results in loss of information, we decided to use the protein and cDNA sequence covering the first three variable exons for our study. This part covers roughly 40% of the total *GUCY2* sequence, displaying between 40% and 60% homology among vertebrate protein sequences. To trace the evolution of the *GUCY2* family, with respect to gene losses, duplications and modifications between major taxa, we chose a circular phylogenetic representation separating the groups based on the GC-E phylogeny. In a first survey analysis, we selected one to two commonly used representatives of each major clade. Colored bars illustrate the presence, pseudogenization and/or absence of a particular enzyme (fig. 1). While the entire clade of sauropsids, (birds [e.g., *Gallus gallus*], lizards [e.g., *Anolis carolinensis*], snakes [e.g., *Ophiophagus hannah*], crocodiles [e.g., *Alligator mississippiensis*] and turtles [e.g., *Pelodiscus sinensis*]) lacks a GC-F sequence, mammals display multiple, seeming independent inactivating mutations with no obvious pattern. Some mammals have retained open reading frames for all three *GUCY2* genes, whereas others have accumulated potentially inactivating mutations in either GC-F or GC-D (fig. 1). This is in contrast to teleosts, where some members even increased the number of GC enzymes, suggesting the retention of duplicated *Gucy2* genes might be beneficial.

Within the phylogenetic tree visual GCs are not closest relatives, but are both more closely related to olfactory GC-D (supplements S2, S7, S10–S12, Supplementary Material online). Nevertheless, all the three different GCs in mammals and fish group within a common branch showing closest relations to each other. Synteny analysis confirmed our nomenclature and phylogenetic relation (GC-E supplement S3, GC-F supplement S4, and GC-D supplement S5, Supplementary Material online), which is in contrast to the nomenclature and grouping found in the ensembl database where teleosts have no homolog to the human GC-F.

### Diversity of Visual GCs Is Reflected in the Size of the First Exon

Vertebrate species predating 2R WGD had one common ancestor of GC-D/E/F. The GC-E and GC-F/D split seems to have occurred before the vertebrate-specific WGD as studies in lamprey and hagfish have demonstrated (Lamb and Hunt 2018). Initially, following a WGD event, the exon–intron structure of the duplicated genes is indistinguishable. Most often duplicated genes, being relieved from selective pressure, become nonfunctionalized, subsequently pseudogenized and eventually eliminated from the genome. Sometimes duplicated genes undergo subfunctionalization, meaning that they start sharing the ancestral function of the gene. During subfunctionalization, regulatory elements are modified giving each duplicated gene a specific expression pattern and therefore a specific localization of action. Rarely duplicated genes can also neofunctionalize, not only influencing the regulatory elements of the duplicated genes, but also altering their function (for review see [Glasauer and Neuhauss 2014]). Most regulatory elements are not only found upstream of the ATG start codon, but can also be located in intronic regions, especially within those close to the transcriptional start site (UTR introns and the one following the transcriptional start site) (for review see [Hernandez-Garcia and Finer 2014]). Interestingly, in the case of therian species, which have both visual enzymes, the first intron in *Gucy2e* is ∼100 bp, whereas the first intron in *Gucy2f* is ∼100 times larger (supplement S6, Supplementary Material online). This implies that the first *Gucy2f* intron may contain cis regulatory elements that influence and/or fine tune expression. In contrast to this, in sauropsids, which have lost the second visual guanylyl cyclase, the size of the first intron of the remaining *Gucy2E* is with 1,500 bp somewhere in between the *Gucy2E* and *Gucy2F* situation in theria. This suggests that gene regulation in sauropsids may involve elements of both visual cyclases.

### *Gucy2* Gene Inactivation in “Bottleneck Species” Can Affect Entire Clades

**Fig. 2.**
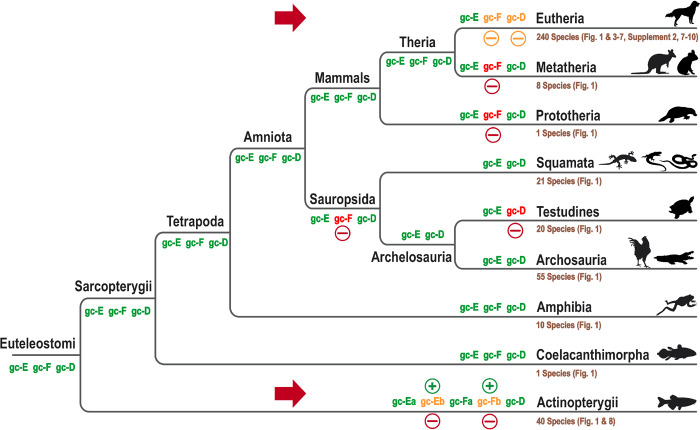
GC gene inactivation pattern in bony vertebrates. A cladogram, starting at the euteleostomians, including all major vertebrate orders, is shown. Based on presence or absence of GC genes in downstream clades the minimal GC gene content of a common ancestor at a branch point is given in green (present) or red (absent) letters. First occurrences of gene inactivation and duplication are highlighted by red minus signs or green plus signs, respectively. Red Arrows indicate orders in which no consistent gene loss can be observed. Enzymes that show an inconsistent pattern are depicted in orange. Number of analyzed species in each order and figures with additional details are given in brown. For readability, pictograms of typical representatives of each order are included. Note that four major inactivation events influenced the entire downstream clade. As the common ancestor of birds, reptiles, and turtles has lost an intact GC-F enzyme all descending species in this clade lack this enzyme. Moreover, following the Archosauria/Testudines split, the common ancestor of turtles has lost an intact GC-D variant. Gene inactivation can also be seen at the base of the Metatherian and Prototerian split, leaving both orders without a functional GC-F gene.

In order to systematically reconstruct *Gucy2* gene losses following 2R WGD, we analyzed the minimal gene content at the base of each major clade starting at the *Euteleostomi* level, representing the common ancestor of all bony vertebrates (fig. 2). Within both major subbranches of *Eutelestomi*, namely ray fined (*Actinopterygii*) and lobe fined fishes (*Sarcopterygii*; including all tetrapods), unmutated representatives for all three GC enzymes can be found. This supports the interpretation that the common ancestors must have had a full complement of GC (GC-E, GC-F, and GC-D). This pattern persists at the base of amniotes, which subsequently split into mammals and sauropsids (reptiles, birds, turtles, and crocodiles). In the mammalian clade, we find species having either intact cDNAs for all three cyclases or displaying mutations in GC-F or GC-D (fig. 2), whereas all analyzed sauropsids completely lack a GC-F sequence. This indicates that the common ancestor of all lizard-faced animals had already lost this enzyme. Consequently, all descending species lack this second visual GC. Despite this fact, color vision and visual acuity of birds and reptiles is at least comparable to other visual species in the animal kingdom, suggesting that species with only one visual GC do not necessary have decreased visual abilities. One possible explanation is that gene inactivation of *Gucy2F* in sauropsids, which already occurred around 320 Ma (Blair and Hedges 2005), gave modern birds and reptiles enough time to compensate GC-F loss by visual adaptations and/or functional expansion of the GC-E enzyme. A similar argument can be made for the loss of *Gucy2D* in all modern turtles. Following the *Testudine* (turtles)/*Archosauria* (birds and crocodiles) split, which took place ∼260 Ma (Wang et al. 2013), the common ancestor of all turtles must have readily lost the olfactory GC-D enzyme. Ray finned fishes are the most diverse subclass with active *Gucy2D/E/F* gene numbers ranging from two to six.

In the eutherian subbranch, the accumulation of inactivating mutations occurred several times independently. This indicates that requirements for specific visual or olfactory needs might have shaped the *Gucy2* gene content. Since knockdown of *Gucy2e* in mice has a drastic effect on vision and cone photoreceptor survival, it is hardly surprising that among all 300 analyzed species we did not find a single one lacking *Gucy2e*. Hence, GC-E serves as the primary visual GC and its function is necessary as well as sufficient to cover visual requirements.

This raises the question why o so many species retain a *Gucy2f* gene, when GC-E alone seems to be sufficient? Similarly, what are the specialized functions of the olfactory GC-D enzyme, as some species still use it, whereas others are perfectly viable without it? In the following paragraphs, we try to shine light on these questions and find an evolutionary and ecological rational by revealing possible patterns of gene loss and retention.

### Does *Gucy2d* Gene Retention Correlate with Socially Transmitted Odor Information?

**Fig. 3.**
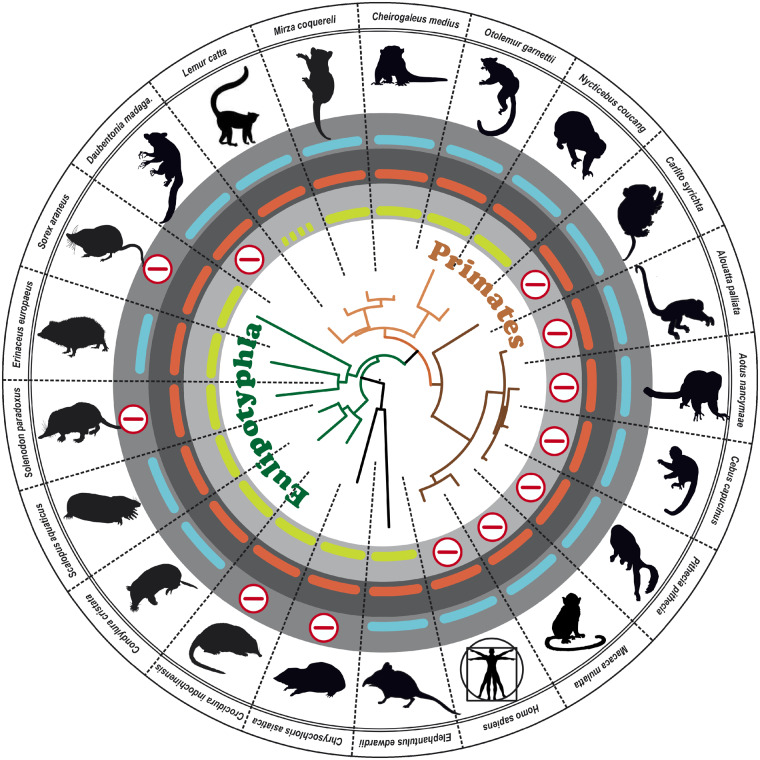
GC gene loss in primates and *Eulipotyphla*. In the center the GC-E phylogeny of the analyzed primates (brown) and *Eulipotyphla* (green) species is shown. Pictograms for each chosen representative are shown and scientific names are given. The presence of intact GC-E (red), GC-F (blue), and GC-D (light green) is indicated by corresponding bars. Absent or inactivated genes are highlighted by an encircled red minus sign. Note that all dry-nosed primates (haplorhini) that also includes tarsiers (red asterisk), lack an intact GC-D enzyme. In contrast, wet-nosed primates, with the exception of the aye-aye, still use the GC-D enzyme. Interestingly, some lemurs seem to show first signs of GC-D pseudogenization as some rare inactivating mutations start appearing in some species (represented by the dotted bar). Interestingly, being nocturnal or living underneath the earth has not prevented some *Eulipotyphla* from retaining a second (GC-F) visual cyclase. Excitingly, the only blind species of afrotherians lacks the second visual guanylyl cyclase GC-F.

We took a closer look at several mammalian orders, as gene inactivation and preservation had occurred multiple times independently in this clade (fig. 3). In primates, *Gucy2d* is frequently found as inactivated pseudogene or absent all together (Young et al. 2007). Interestingly, members of the colloquially called wet-nosed monkeys (*Strepsirrhini*) including *Lemuriformes* (lemurs of Madagascar) and *Loriformes* (loris and galagos) often still possess *Gucy2d* genes with an intact open reading frame. Wet-nosed primates have a very highly developed sense of smell (Gilad et al. 2004). Dry-nosed monkeys (*Haplorhini*), on the other hand, have reduced sensitivity to pheromones and a less well-developed sense of smell. GC-D positive neurons in mice are involved in socially transmitted food preference (STFP), by detecting odors present in the urine and feces (Leinders-Zufall et al. 2007; Munger et al. 2010). Such a concept may also apply for *Strepsirrhini*. Interestingly, some wet-nosed monkeys have started to accumulate inactivating mutations in *Gucy2d*. While loris (e.g., *Nycticebus coucang*) and galagos (e.g., *Otolemur garnettii*) clearly retained *Gucy2d* version with an intact open reading framein some lemurs partial pseudogenization of the gene is ongoing (fig. 3, supplement S7, Supplementary Material online), suggesting that their need to detect certain GC-D dependent stimuli is no longer relevant. The presence of inactivating mutations in GC-D in the aye-aye (*Daubentonia madagascariensis*) is rather surprising as this species utilizes urine scent marks. Remarkably, all the frame shift and nonsense mutations in *Strepsirrhini* detected so far are within the first exon, making it possible that the use of alternative start sites might still lead to a functional protein. The absence of *Gucy2d* correlates with the absence of a rhinarium, a furless skin surface around the nostrils that is often considered an additional sense organ, transmitting pheromones or other scent marked odors to the vomeronasal organ (VNO). However, so far no expression of GC-D in the VNO has been described, leaving it an open question whether the rhinarium may be a way to transmit signals involving the GC-D enzyme.

### Multiple *Gucy2* Gene Loss Occurs in Ungulates, Aftotherians, Carnivores, and Rodents

As the pattern of gene preservation and/or gene loss is not evident in the hitherto analyzed mammalian orders, we extended our analysis to additional mammalian clades. In the quite diverse afrotherian and ungulate subgroups, active genome reshaping in respect to *Gucy2*s is ongoing (fig. 3, supplements S8 and S9, Supplementary Material online). In carnivores, our analysis showed that cat-like carnivores (*Feliformia*) as well as dog-like carnivors (*Carniformia*) generally retained a full GC content. In contrast, weasel-like carnivores (*Mustelidae*; supplement S10, Supplementary Material online) and mangoose (*Herpsetidae*; supplement S10, Supplementary Material online), which belong to different clades, but occupy similar ecological niches, have both independently accumulated inactivating mutations in the second visual GC (*Gucy2f*). As many of these animals still rely on vision, this may very well be due to GC-F inactivation in a common ancestor, hence all descending species had to adapt to this.

**Fig. 4.**
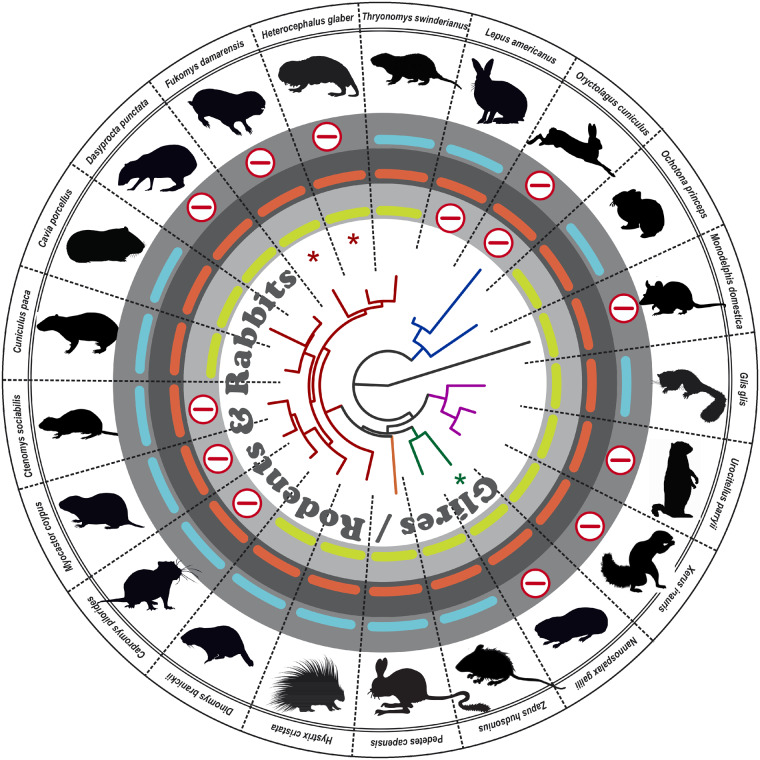
Rodents and rabbits show multiple independent GC gene losses. In the center, the GC-E phylogeny of the studied squirrel-like (violet), mouse-like (green), springhares (red-brown), porcupine-like (ruby red), and rabbit-like (blue) species of rodents and rabbits is shown. Pictograms for each chosen representative are shown and scientific names are given. The presence of intact GC-E (red), GC-F (blue), and GC-D (light green) is indicated by corresponding bars. Absent or inactivated genes are highlighted by an encircled red minus sign. Note that all visually impaired subsurface living species (asterisks) lack the second visual guanylyl cyclase GC-F. However, also some diurnal species, such as squirrels, rabbits and agoutis seem to make do without a functional GC-F enzyme.

Also glires, which comprise the orders of rabbits and rodents covering ∼2,500 different species (Wilson and Reeder 2007), show some remarkable alterations. In the *Myomorpha* family (mouse-like rodents, highlighted in green and represented by *Zapus hudsonius* in fig. 4) all analyzed species (*n* = 15) have retained intact sequences of all three *Gucy2*s. The notable exception is the blind mole rat (*Nannospalax galili*), which has accumulated several inactivating mutations in GC-F. In the *Hystricomorpha* clade (porcupine-related rodents, highlighted in ruby red in fig. 4) more than half (12 out of 18 sampled species) no longer have unmutated *Gucy2f* or *Gucy2d* sequences. The *Gucy2d* sequences of the three phylogenetically closely related species of hutia (*Capromys pilorides*), tuco (*Ctenomys sociabilis*), and nutria (*Myocastor coypus*) do not code for a functional olfactory GC-D, whereas the *Gucy2f* cDNA of the American agouti (*Dasyprocta punctate*) accumulated several inactivating mutations (fig. 4). In the *Lagomorpha* order (rabbit-like species including hares, rabbits, and pikas), the number of intact GC’s varies most. Although in pikas (*Ochotona princeps*) all three *Gucy2* sequences code for active enzyme variants, hare (e.g., *Lepus americanus*) and rabbit genomes no longer display a sequence coding for the GC-D enzyme. In addition, rabbits (e.g., *Oryctolagus cuniculus*) only have a *Gucy2f* pseudogene, relying completely on GC-E as the sole functional enzyme. Within the suborder of the squirrel-like *Scioromorpha*, dormice (e.g., *Glis glis*) retain open reading frames sequences for all three GCs, whereas all family members of the *Sciuridae* (encompassing amongst others, tree and ground squirrels [e.g., *Xerus inauris*], chipmunks, marmots [e.g., *Urocitellus parryii*], and prairie dogs) lack a nonmutated GC-F variant (fig. 4). Interestingly, visually impaired rodents that primarily live underground all display multiple inactivating mutations in Gucy2f, leading to inactive GC-F variants (asterisks).

### Regressive Evolution in Subterranean Species Leads to Adaptations in GC-F Usage

The eyes and visual capabilities of subterranean species analyzed in this study are clearly degenerated, leaving these species with only rudimentary vision and eyes. These species are polyphyletic and have adapted to underground life by convergent evolution. Moles and mole rats that occupy a subterranean niche, phylogenetically grouped into three different clades. *Bathyergidae* (porcupine-like rodents) include *Heterocephalus glaber* and *Fukomys damarensis*, *Myomorpha* (mouse-like rodents) *Nannospalax galili* (fig. 4), *Afrotheria* include *Chrysochloris asiatica* (fig. 3), and *Eulipotyphla* (insect eaters) include *Condylura cristata*, *Scalopus aquaticus*, and *Uropsilus gracilis* (fig. 3).

Despite their adaptation to subterranean life and their different evolutionary origin, all these species retain a functional GC-E visual cyclase. This demonstrates that even species with rudimentary vision retain at least one visual GC enzyme. This is hardly surprising as retaining a functional visual transduction cascade, even if only for detecting changes in illumination, still requires at least one active component of each protein involved in the visual transduction cascade. Moreover, as GCs have been shown to be of structural importance to the retina, by forming a complex with other visual proteins (Baehr et al. 2007; Karan et al. 2010), the complete developmental program of eye and head might be compromised without a functional GC enzyme.

However, out of the seven visually impaired species, the star-nosed mole (*C. cristata*) and the eastern mole (*S. aquaticus*) show no obvious accumulation of inactivating mutations, challenging the assumption that being nearly blind releases the second visual GC enzyme of selective pressure. There are some possible explanations. Regressive evolution may not have occurred or completed in star-nosed moles (*C. cristata*). In contrast to the monochromatic golden moles (*C. asiatica*), star-nosed moles are functional dichromats and still often leave their underground habitats in search of food above ground (Emerling and Springer 2014). As *Gucy2f* knockdown in mice points to an involvement in the rod system, dim light vision might indeed benefit from a functional GC-F enzyme. This begs the question as to why the eastern mole (*S. aquaticus*), which no longer relies on a sense of vision still seems to have an intact GC-F gene. All other moles seem to be only capable of detecting changes in illuminations and their physiological response to light (measured by electroretinography) is greatly reduced. These rudimentary visual functions seem to help them leaving their burrows in cases of emergencies and reseal breaches in their tunnel systems (Němec et al. 2008).

At this point it is worth mentioning that some members of the *Eulipotyphla*, namely shrews (e.g., *Sorex araneus* and *Crocidura indochinensis*) and solenodons (e.g., *Solenodon paradoxus*), also accumulated a number of inactivating mutations in *Gucy2f* gene, leading to an inactive GC-F enzyme (fig. 3). As shrews in general have relatively poor vision but excellent senses of hearing and smell it is plausible that the second visual enzyme GC-F is no longer under selective pressure. Moreover, shrews have developed an additional possibility to explore the immediate environment, namely echolocation (Forsman and Malmquist 1988; Siemers et al. 2009; Sanchez et al. 2019), making them less dependent on vision.

### Method of Food Location Determines the Activity of Visual and Olfactory GC in Bats

**Fig. 5.**
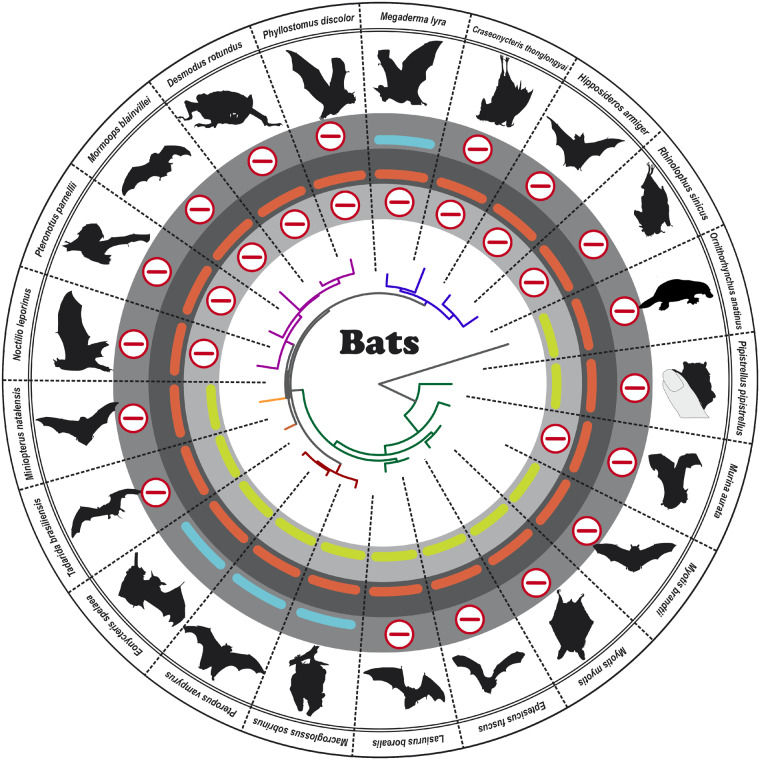
Extensive guanylyl cyclase gene loss occurs in the order of bats. In the center, the GC-E phylogeny of the analyzed bats is shown. Different suborders and families are highlighted in colors. Flying foxes (Megabats) are shown in ruby red, common bats (vespertilionidae) in green, horseshoe-nosed bats in blue, leaf-nosed-like bats in purple, free-tailed bats in red-brown, and longwinged bats in orange. Pictograms for each chosen representative are shown and scientific names are given. The presence of intact GC-E (red), GC-F (blue), and GC-D (light green) is indicated by corresponding bars. Absent or inactivated genes are highlighted by an encircled red minus sign. Note that nonecholocating species (ruby red) kept active variants of the second visual cyclase GC-F. Interestingly, horseshoe- and leaf-nosed-like bats further lack GC-D.

As the variations in the clade of rodents and rabbits are quite remarkable, we extended out detailed analysis to further orders, including bats and teleosts. Bats are, after rodents, the second largest order of mammals with >1,200 different species (Wilson and Reeder 2007). Most species are either frugivores, nectarivores or insectivores, but some species also prey on small animals or use blood as their primary diet ((Findley 1993) for review see (Sotero-Caio et al. 2017)). Although bats are commonly nocturnal, members of the megabat family (Pteropodidae) have nocturnal, crepuscular and a few diurnal species (Melin et al. 2014). Megabats have comparatively large eyes, are primarily frugivores and/or nectarivores and only few species use echolocation for foraging. They mainly rely on their well-developed visual (Wang et al. 2004) and olfactory systems (Luft et al. 2003). Nevertheless, being specialized to low light conditions, color vision in *Pteropodidae* is diminished with most family members being monochromats or dichromats. Rod density in the retina of megabats is highly adapted to twilight vision. Therefore, it is hardly surprising that both visual GCs genes lack any inactivating mutations in megabats (fig. 5).

In contrast to megabats, microbats (including common bats [*Vespertilionidae*], leaf-nosed bats [*Phyllostomidae*], horseshoe bats [*Rhinolophidae*], free-tailed bats [*Molossidae*], long-winged bats [*Miniopteridae*], bulldog bats [*Noctilionidae*], hog-nosed bats [*Craseonycteridae*], and mustached bats [*Mormoopidae*]), use mainly echolocation and/or olfaction for food detection (Gonzalez-Terrazas et al. 2016). Most microbats are insectivores hunting during night hours. Consequently, eyes of microbats are commonly rudimentary, serving merely to detect changes in illumination (Rydell and Eklöf 2003; Liu et al. 2015). As already discussed in the cases of moles, even rudimentary vision requires a functional visual transduction cascade. As the main GC in the visual system is GC-E, *Gucy2f* became pseudogenized in most microbat species (fig. 5). An interesting exception is the microbat *Megaderma lyra* (greater false vampire bat), which has retained both visual GCs. This can be rationalized by *M. lyra* having exceptional large eyes and therefore presumably relying on visual input for foraging (Ratcliffe and Ter Hofstede 2005).

In the case of leaf-nosed (*Phyllostomidae*; fig. 5 purple) and horseshoe bats (*Rhinolophidae*; fig. 5 blue), which have a number of frugivores, nactarivores, and omnivores amongst them, smell-based food localization cannot be mediated by the GC-D system, as all members of this family only have Gucy2D pseudogenes leading to nonfunctional GC-D variants.

It has been recently shown, that in contrast to mice where STFP via the GC-D system has been observed, mouth to mouth feeding of pups in the bat species *Glossophaga soricina* (a member of the leaf nosed-bats) has no effect on food preference during adulthood (Rose et al. 2019). Although having a functional GC-D might have been beneficial in locating CO_2_ released by rotten food, detecting regular odors does not require GC-D, as these volatiles are detected by the adenylyl cyclase system located in the main OE. Surprisingly, common bats (*Vespertilionidae*) that are mainly insectivores have retained an active GC-D variant, suggesting that some aspect of GC-D dependent olfaction is still in use. Big brown bats (*Eptesicus fuscus*) uses chemicals in a social context for colony mate recognition, preferring individuals from their own roost (Bloss et al. 2002).

### Adaptation to Aquatic Life in Fish Seems to Require Intact Variants of All Three GCs

Fish represent a paraphyletic group including cartilaginous (*Chondrichthyes*) and bony (*Osteichtyes*) fishes. Although the class of cartilaginous fish can be subdivided into two subclasses, namely the shark, ray, and skate-like *Elasmobranchii*, and the chimera type *Holocephali*, the superclass of bony fish are formed by the classes of ray-finned fish (Actinopterygii) and lobe-fined fish (Sarcopterygii). With >34,000 different species, ray-finned fish represent the dominant class of vertebrates (www.fishbase.org). Most lobe-fined fish evolved into tetrapods comprising mammals, reptiles and amphibians, leaving only eight extant fish species in this class. Fish have colonized almost all habitats in fresh and seawater. The sensory systems, especially vision and taste/smell of fish are highly developed and visual capabilities of diurnal fish are at least as good as that of mammals (for review see [Caves et al. 2018]). Moreover, several types of fish have an exceptional sense of smell. For instance, salmons discriminate their origin of birth based on characteristic smells of individual rivers (Dittman and Quinn 1996). Depending on their habitats, fish vision has adapted remarkably. Some deep sea fish have extended their repertoire of rhodopsin genes from typically one variant to up to 38 different variants (Musilova et al. 2019) and many diurnal fish use ultraviolet light, which helps in foraging and mate selection (Shi and Yokoyama 2003; Novales Flamarique 2013).

**Fig. 6.**
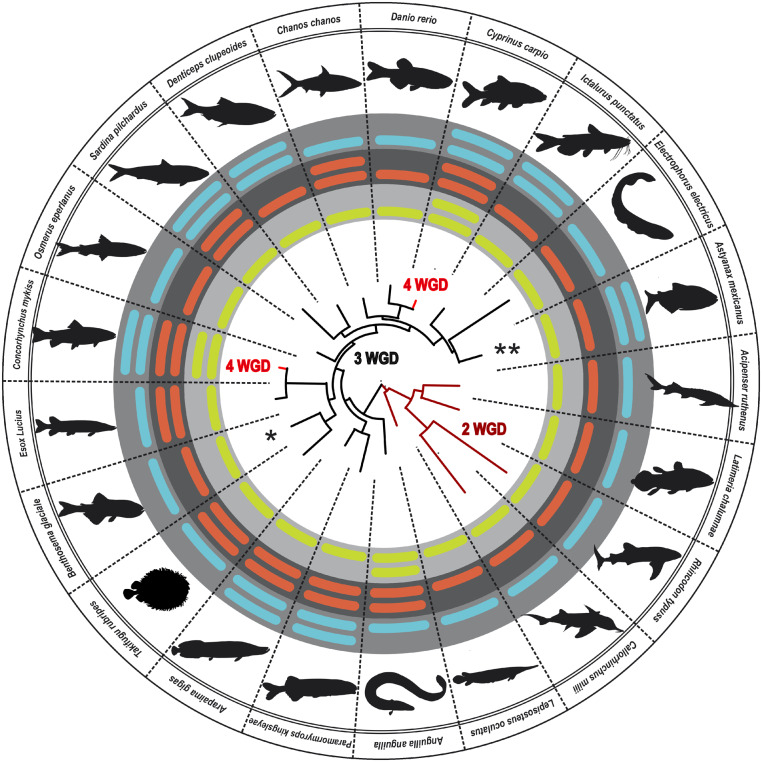
A minimum of two visual and one olfactory guanylyl cyclases is present in all fish species. In the center, the GC-E phylogeny of the paraphyletic group of fish is shown. Branches of ancient fish species not going through the teleost-specific WGD are colored in ruby red (2 WGD), branches of teleosts which underwent 3 WDG are given in black, and branches of species that had 4 WGD are highlighted in red. Pictograms for each chosen species are shown and scientific names are given. Corresponding bars indicate the presence of intact GC-E (red), GC-F (blue), and GC-D (light green). The asterisk marks the deep sea species *Benthosema glaciale* and the double asterisk highlights the blind cave fish *Astyanax mexicanus*. Note that all analyzed fish species have at least one intact variant of the three different guanylyl cyclases and that despite the teleost-specific WGD several species have already nonfunctionalized one of the duplicated paralogs.

Although all vertebrates have gone through 2R WGD, teleost fish have had an additional WGD ∼320 Ma and salmon-like (∼80 Ma) and members of the carp family (∼10 Ma) even went through a fourth round of WGD (for review see [Glasauer and Neuhauss 2014]). Nevertheless, in the case of the zebrafish ∼26% of the duplicated genes were retained following redeploidization (Howe et al. 2013). This demonstrates that sub and/or neofunctionalization events are quite rare and that most duplicated genes are nonfunctionalized during evolution. Although all ancient fish species predating the teleost-specific WGD (fig. 6, shown in ruby red) have active variants of both visual GCs as well as an intact form of the olfactory *Gucy2d* gene, the number of GCs following WGD varies from species to species. Some species like the deep sea fish *Benthosema glaciale*, the smelt *Osmerus eperlanus*, the electric eel *Electrophorus electricus*, and the zebrafish *Danio rerio* have reduced the number of protein coding GCs to the original number (fig. 6) following redeploidization. Around 75% of the analyzed teleost species have at least three, and 25% even four, visual GCs with open reading frames. This increase in GC gene number is compatible with the notion of subfunctionalization and subsequent tissue-specific expression preventing the timely inactivation of these gene duplicates. With the exception of the European eel *Anguilla anguilla* and species that underwent 4 WGD, no teleost fish has more than one intact *Gucy2d* gene (fig. 6). Whether the retention of an additional GC-D enzyme is an advantage for migratory species (salmon and eel) or whether these species have simply not yet inactivated a redundant gene, remains to be determined. However, remarkably no teleost fish has lost its single active *Gucy2d* gene, suggesting that aquatic life benefits from a functional GC-D enzyme.

## Conclusion

**Fig. 7.**
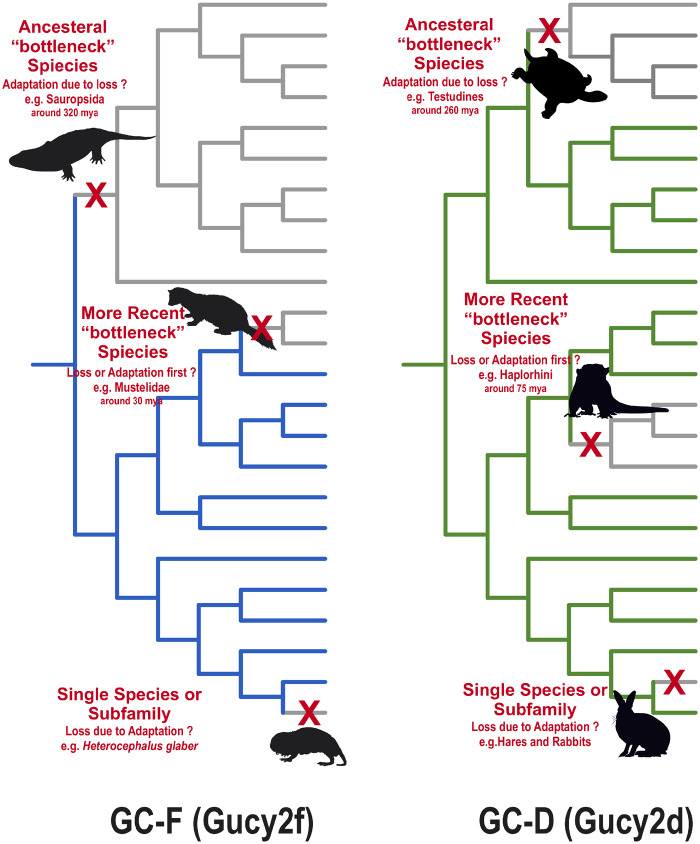
Possible scenarios for GC gene inactivation. Two major possibilities for GC gene loss are given. Approximate time points of the occuring events are indicated and are marked with red crosses. Gene inactivation in ancestral “bottleneck” species is reflected in all descending species (as illustrated by the grey lines in the phylogenetic tree). New traits in all extant spices of this clade had to evolve without these enzymes. Essential functions originally covered by the inactivated gene had to redevelop without it. Over time, alternative possibilities were exploited leading to gene inactivation induced adaptation. In such a scenario, no obvious disadvantages due to gene loss are apparent. On the other hand, recently occurring gene impaiment, only affecting a single species or members of a small subfamily, may be attributes to adaptations in lifestyle and habitat. Here, the gene loses its function, becomes obsolete and subsequently pseudogenized. In this scenario, adaptation to a new lifestyle is the reason for the occurring inactivation. However, several cases in between are also apparent. Gene loss in more recent “bottleneck” species might still be correlated to certain adaptation, like the absence of GC-D in dry-nosed monkeys or the lack of a second visual GC in echolocating night adapted bats. However, sometime descending species have adapted in ways, masking the original reason of gene loss in the ancesteral species (e.g., GC-F loss in weasel-like carnivores [*Mustelidae*] with good diurnal vision).

One of the major driving forces of evolution are genome duplications, enabling sub and/or neofunctionalization of gene ohnologs. Although gene inactivation in ancestral “bottleneck” species can diminish the gene content in an entire clade, regressive, convergent and divergent evolution can lead to gene adaptation in single species or subfamilies (fig. 7). In the case of visual and olfactory guanylyl cyclases different patterns of gene loss are apparent. Gene loss in ancestral bottleneck species has eliminated *Gucy2e* in sauropsides and in addition *Gucy2d* in the testudines clade. Regressive evolution in different species and within different clades has led to multiple *Gucy2e* pseudogenization events in various nonvisual species. *Gucy2d* gene inactivation occurred in species not relying on uroganylin mediated social learning. However, even blind species still express at least one visual GC suggesting that the roles of these enzymes may go beyond its described function in phototransduction.

## Supplementary Material

Supplementary data are available at *Genome Biology and Evolution* online.

## Supplementary Material

evaa192_Supplementary_Data
