# Supplementary material for: Selective Gene Loss of Visual and Olfactory Guanylyl Cyclase Genes Following the Two Rounds of Vertebrate-Specific Whole-Genome Duplications
Source: Genome Biol Evol. 2020 Sep 11;12(11):2153–67. doi: 10.1093/gbe/evaa192 (PMC7674705; doi:10.1093/gbe/evaa192)
Supplement: evaa192_Supplementary_Data [file evaa192_supplementary_data.pdf]

Supplements

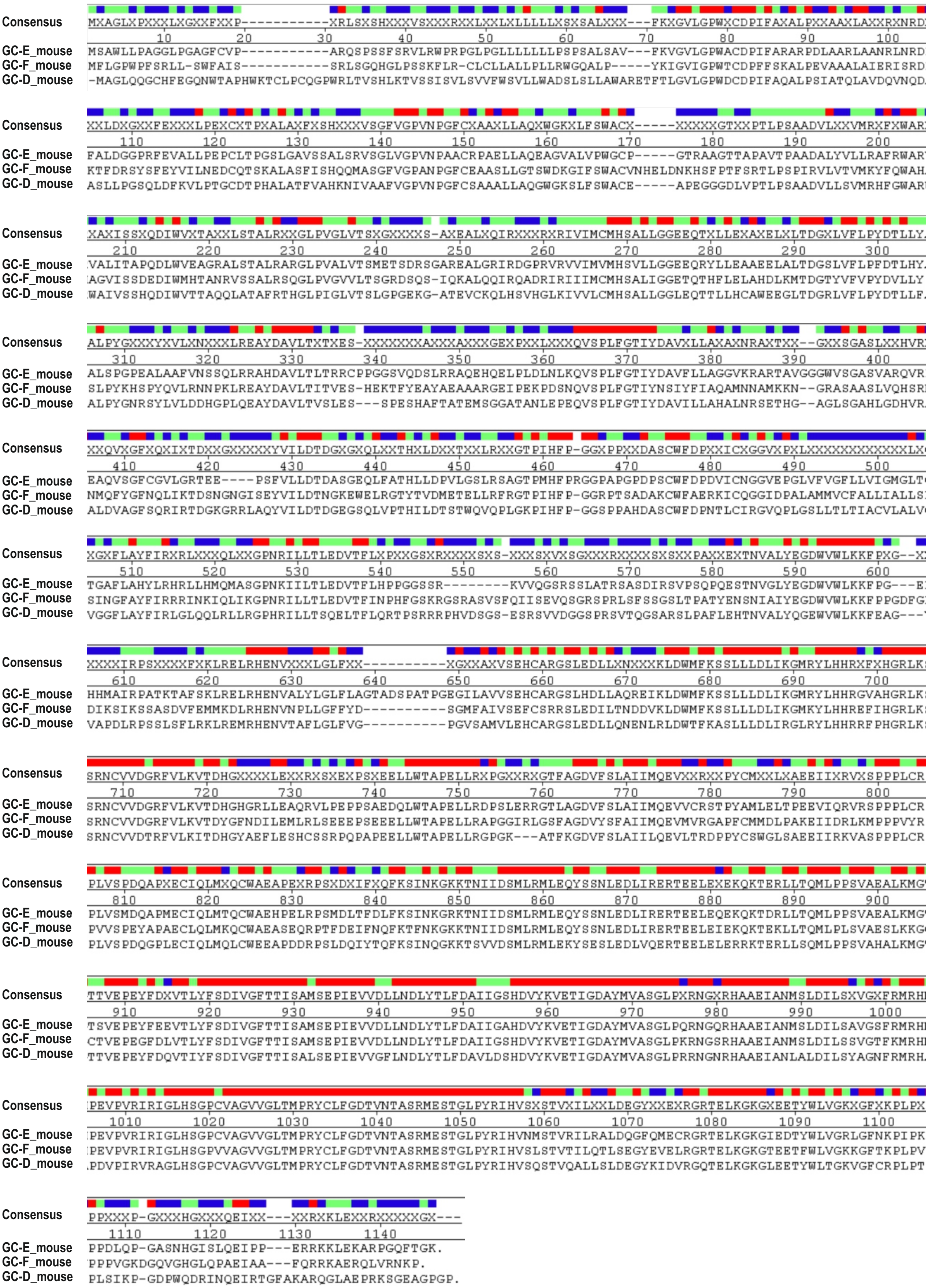

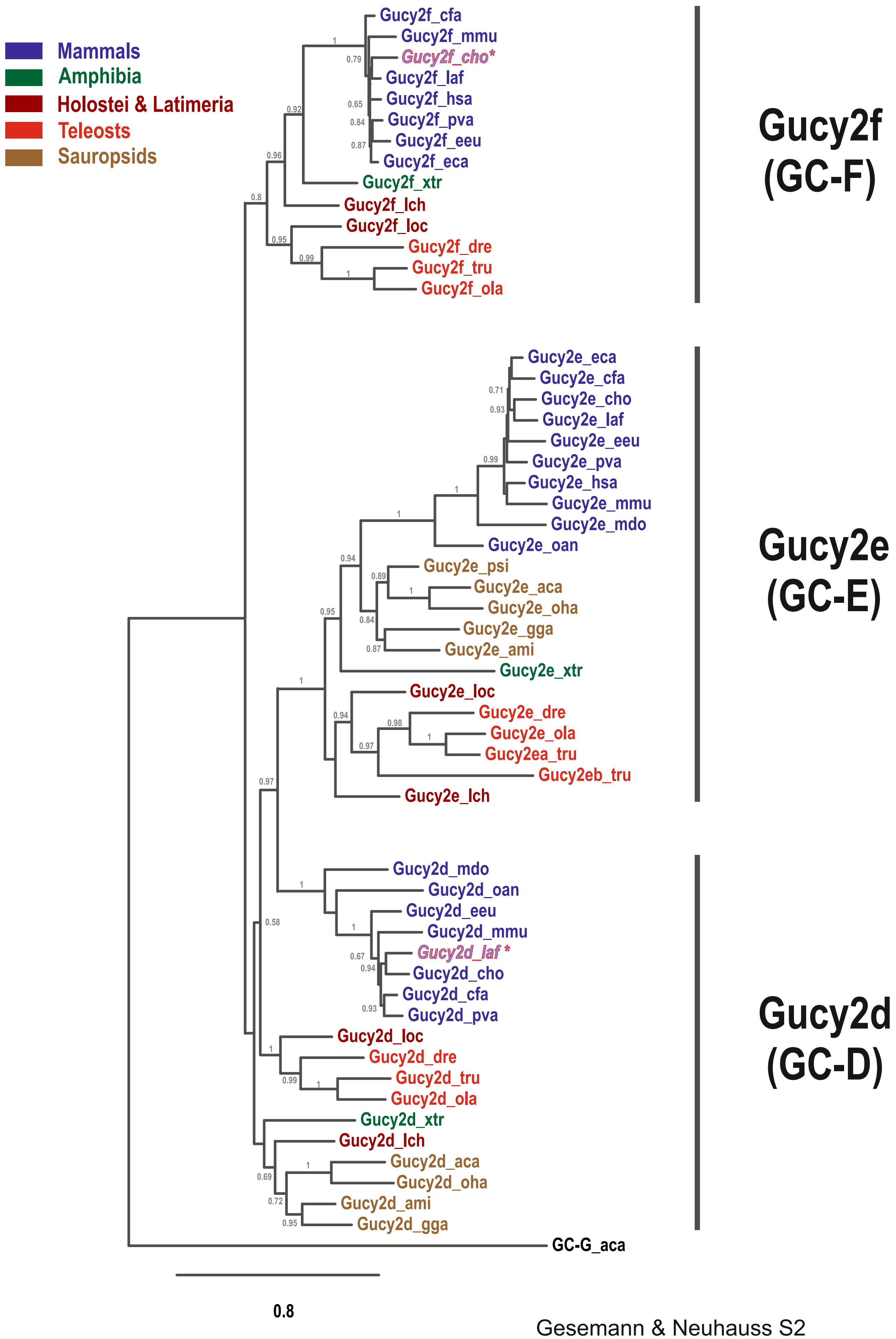

Gesemann & Neuhauss S2

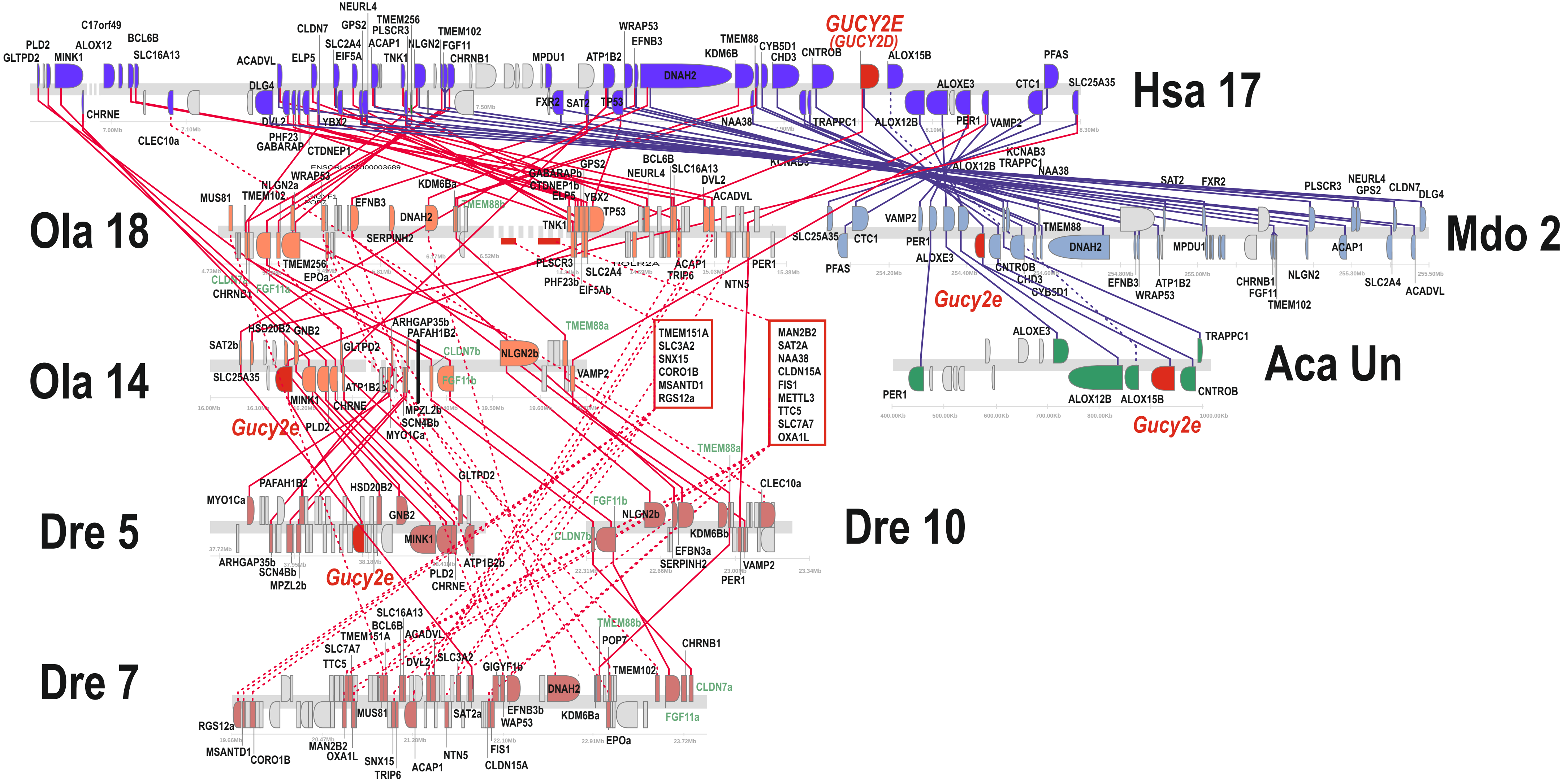

Gesemann & Neuhauss S3

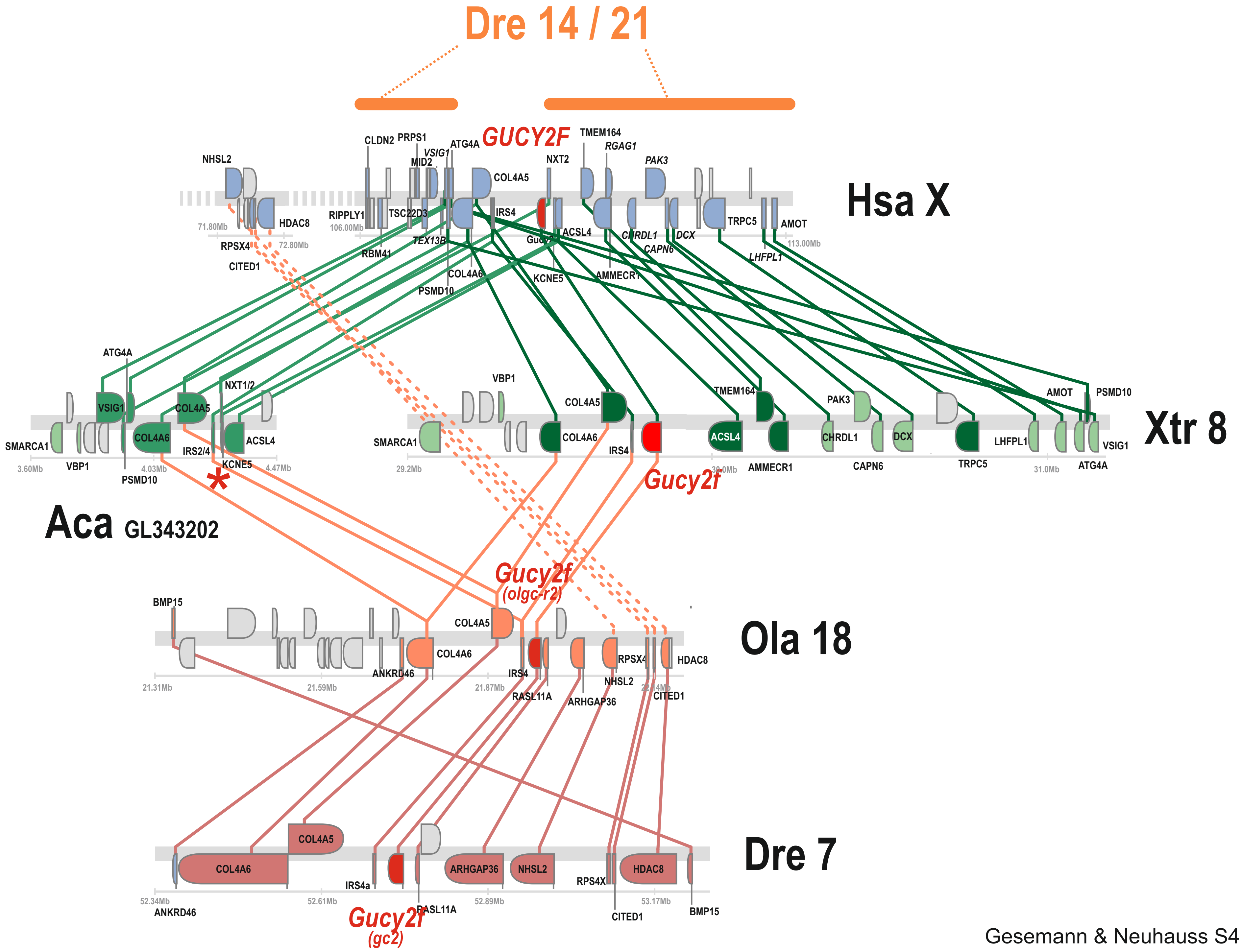

Gesemann & Neuhauss S4

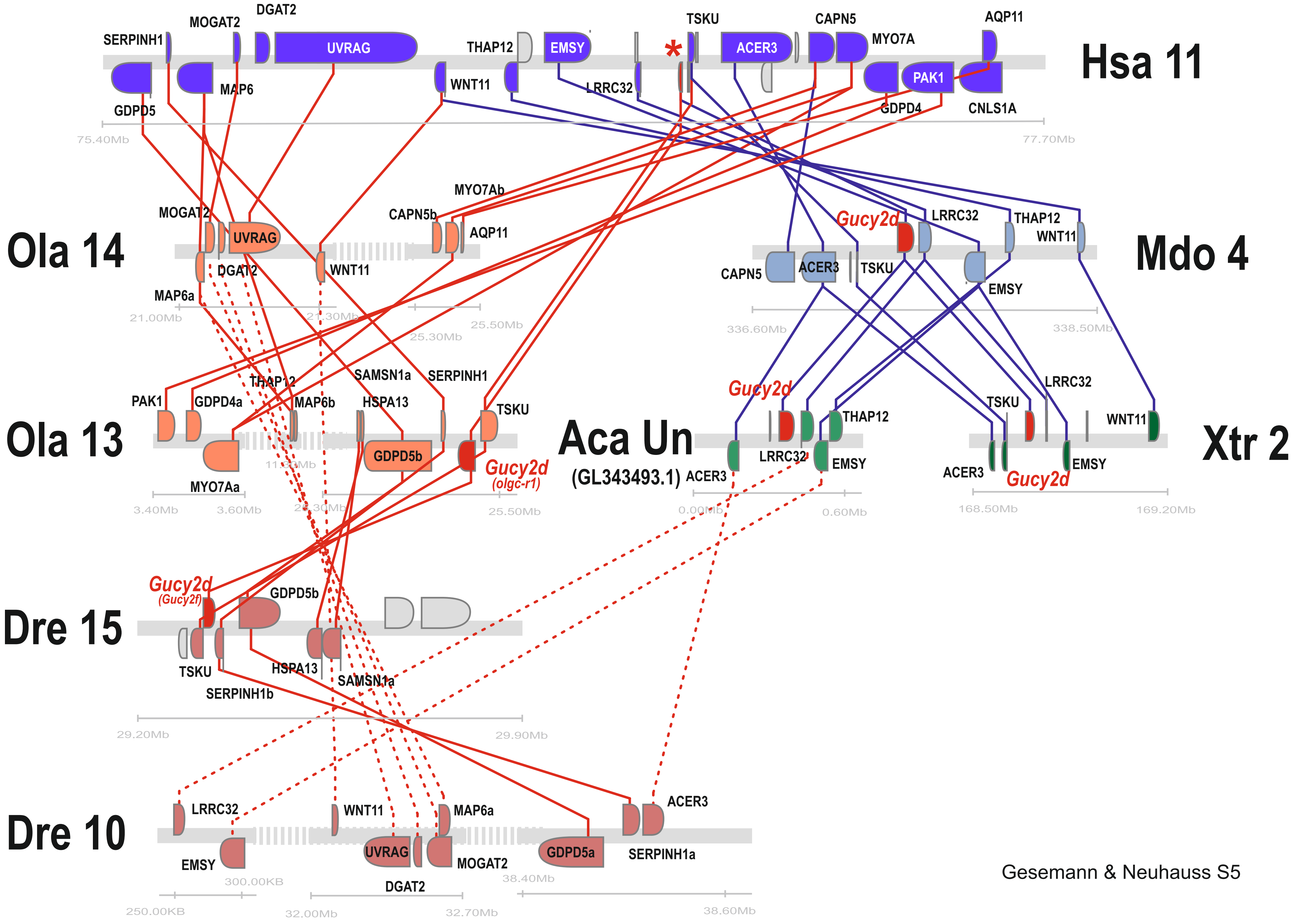

Gesemann & Neuhauss S5

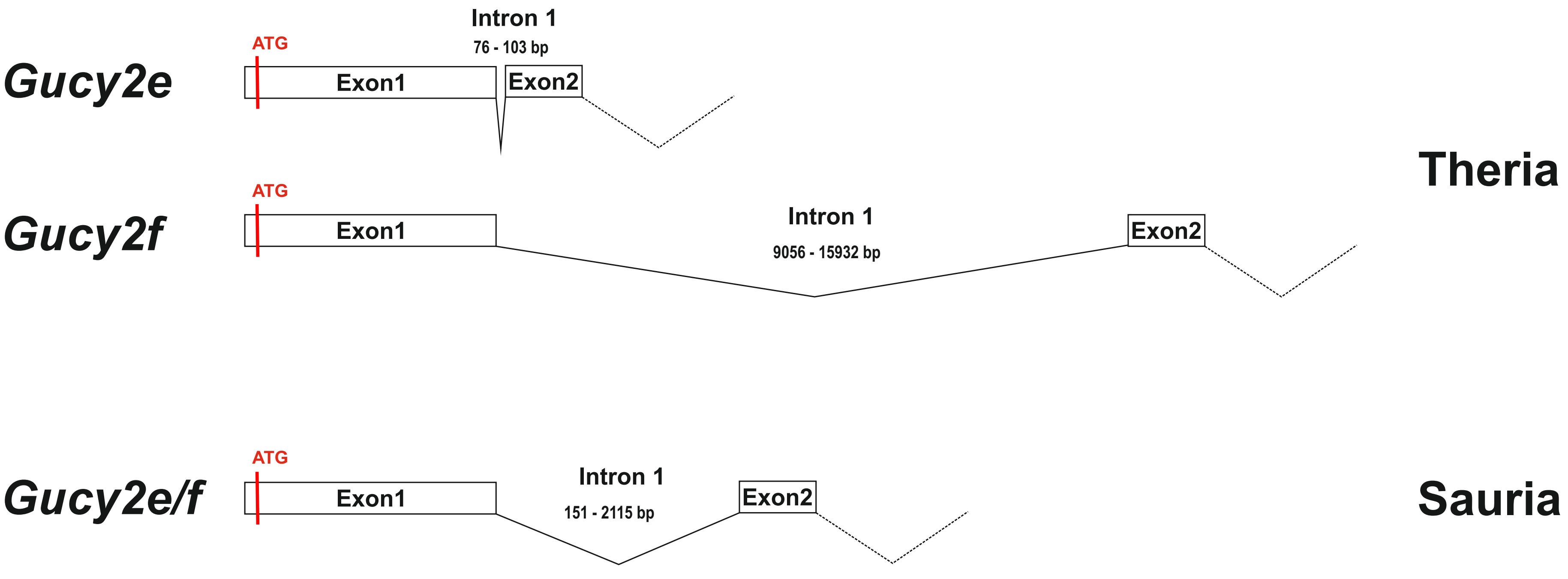

Gesemann & Neuhauss S6

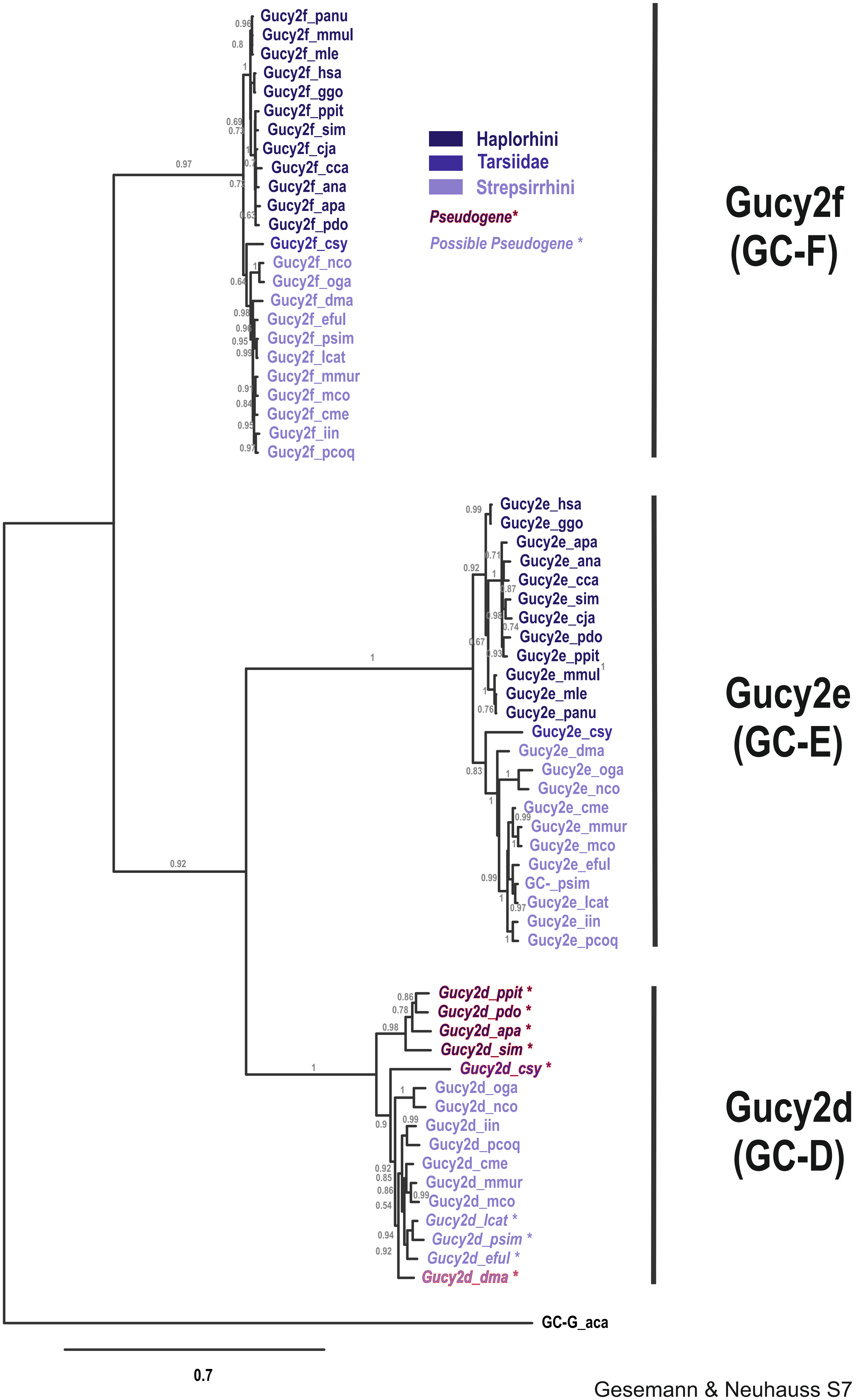

Gesemann & Neuhauss S7

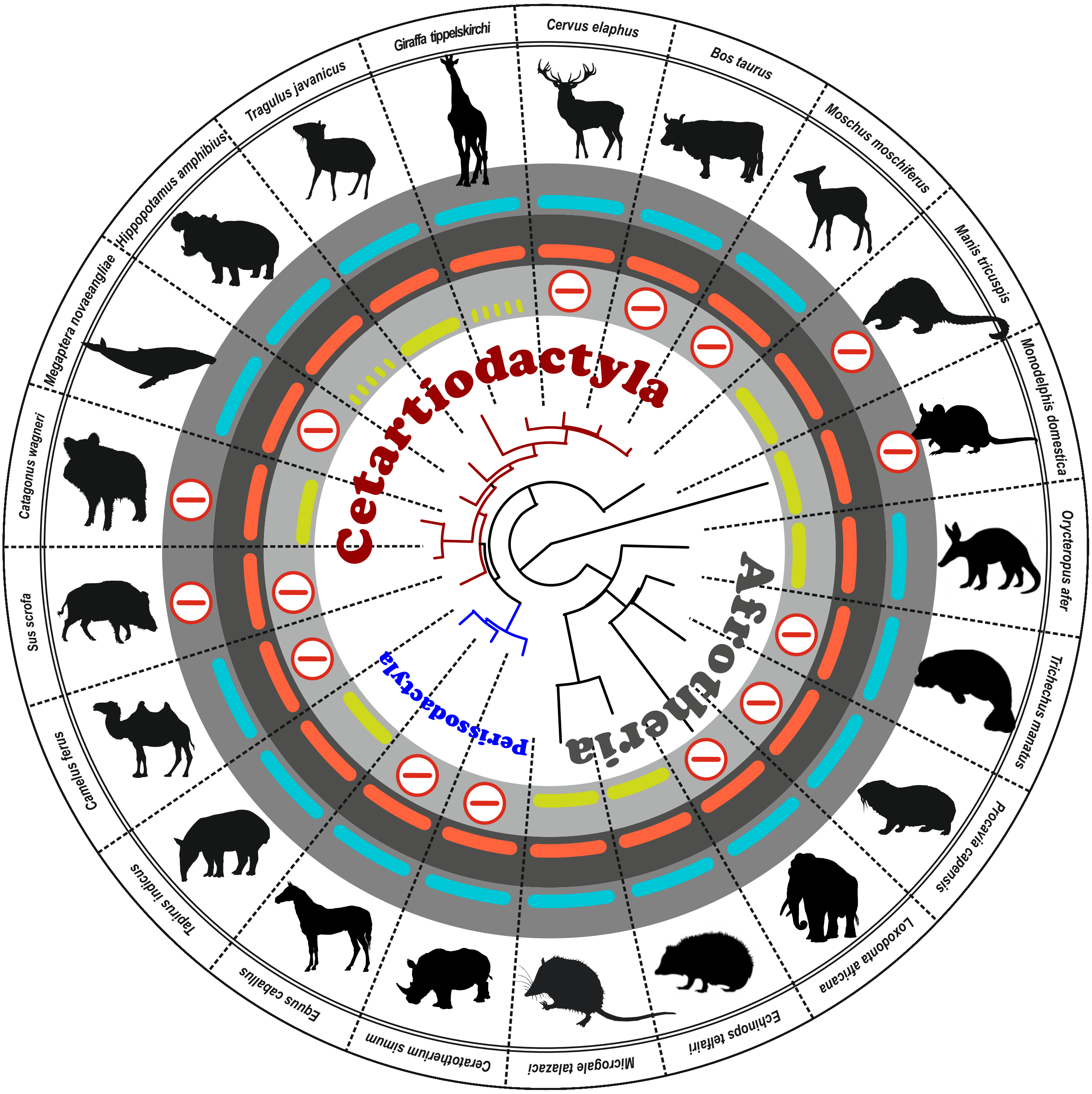
Giraffa tippelskirchi
Cervus elaphus
Bos taurus
Moschus moschiferus
Manis tricuspis
Monodelphis domestica
Orycteropus afer
Trichechus manatus
Procavia capensis
Loxodonta africana
Echinops telfairi
Microgale talazaci
Ceratotherium simum
Equus caballus
Tapirus indicus
Camelus ferus
Sus scrofa
Catagonus wagneri
Megaptera novaeangliae
Hippopotamus amphibius
Tragulus javanicus
Cetartiodactyla
Perissodactyla
Afrotheria

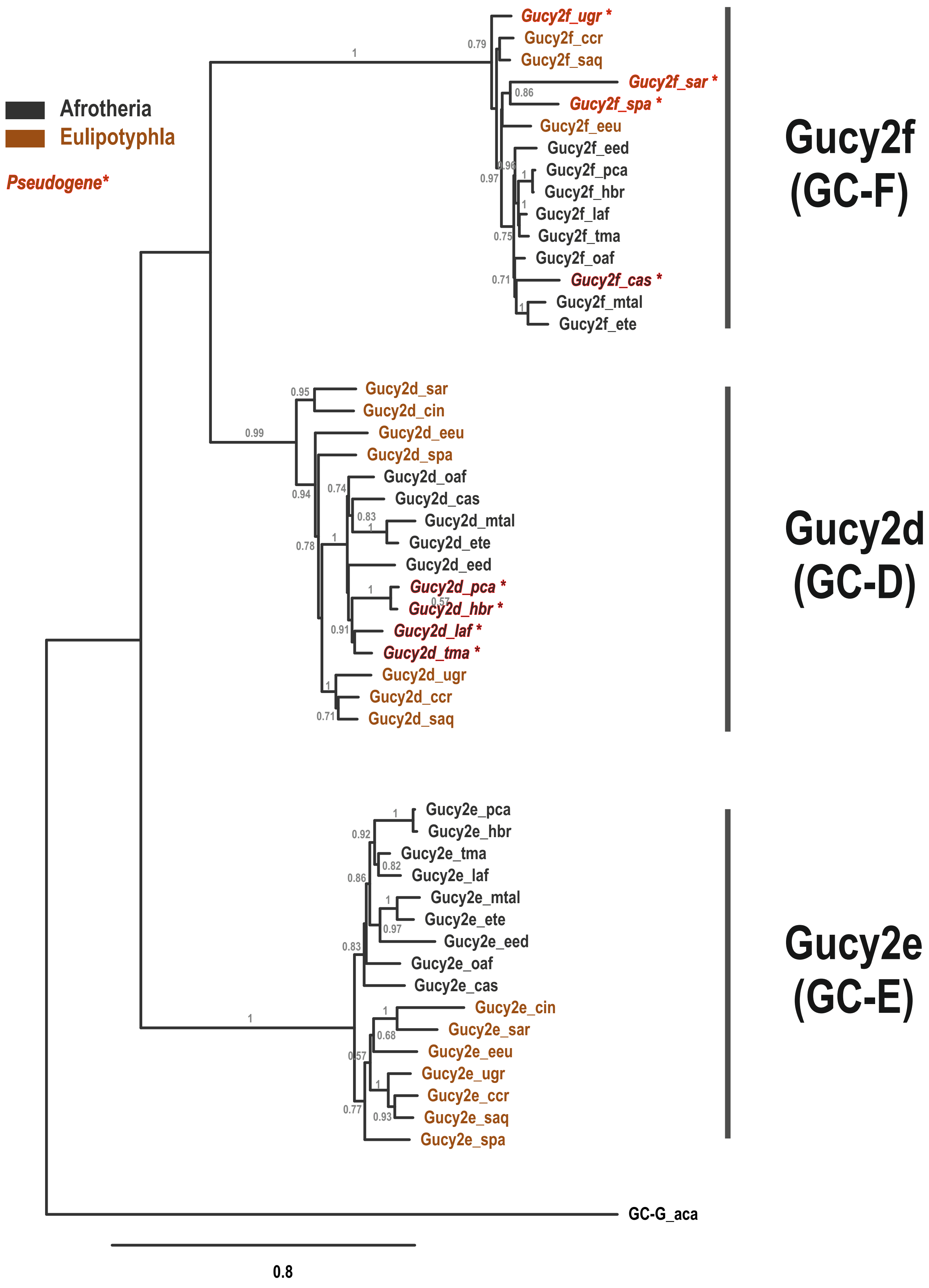
Afrotheria
Eulipotyphla
Pseudogene*
Gucy2f_ugr *
Gucy2f_ccr
Gucy2f_saq
Gucy2f_sar *
Gucy2f_spa *
Gucy2f_eeu
Gucy2f_eed
Gucy2f_pca
Gucy2f_hbr
Gucy2f_laf
Gucy2f_tma
Gucy2f_oaf
Gucy2f_cas *
Gucy2f_mtal
Gucy2f_ete
Gucy2f
(GC-F)
Gucy2d_sar
Gucy2d_cin
Gucy2d_eeu
Gucy2d_spa
Gucy2d_oaf
Gucy2d_cas
Gucy2d_mtal
Gucy2d_ete
Gucy2d_eed
Gucy2d_pca *
Gucy2d_hbr *
Gucy2d_laf *
Gucy2d_tma *
Gucy2d_ugr
Gucy2d_ccr
Gucy2d_saq
Gucy2d
(GC-D)
Gucy2e_pca
Gucy2e_hbr
Gucy2e_tma
Gucy2e_laf
Gucy2e_mtal
Gucy2e_ete
Gucy2e_eed
Gucy2e_oaf
Gucy2e_cas
Gucy2e_cin
Gucy2e_sar
Gucy2e_eeu
Gucy2e_ugr
Gucy2e_ccr
Gucy2e_saq
Gucy2e_spa
Gucy2e
(GC-E)
GC-G_aca
0.8

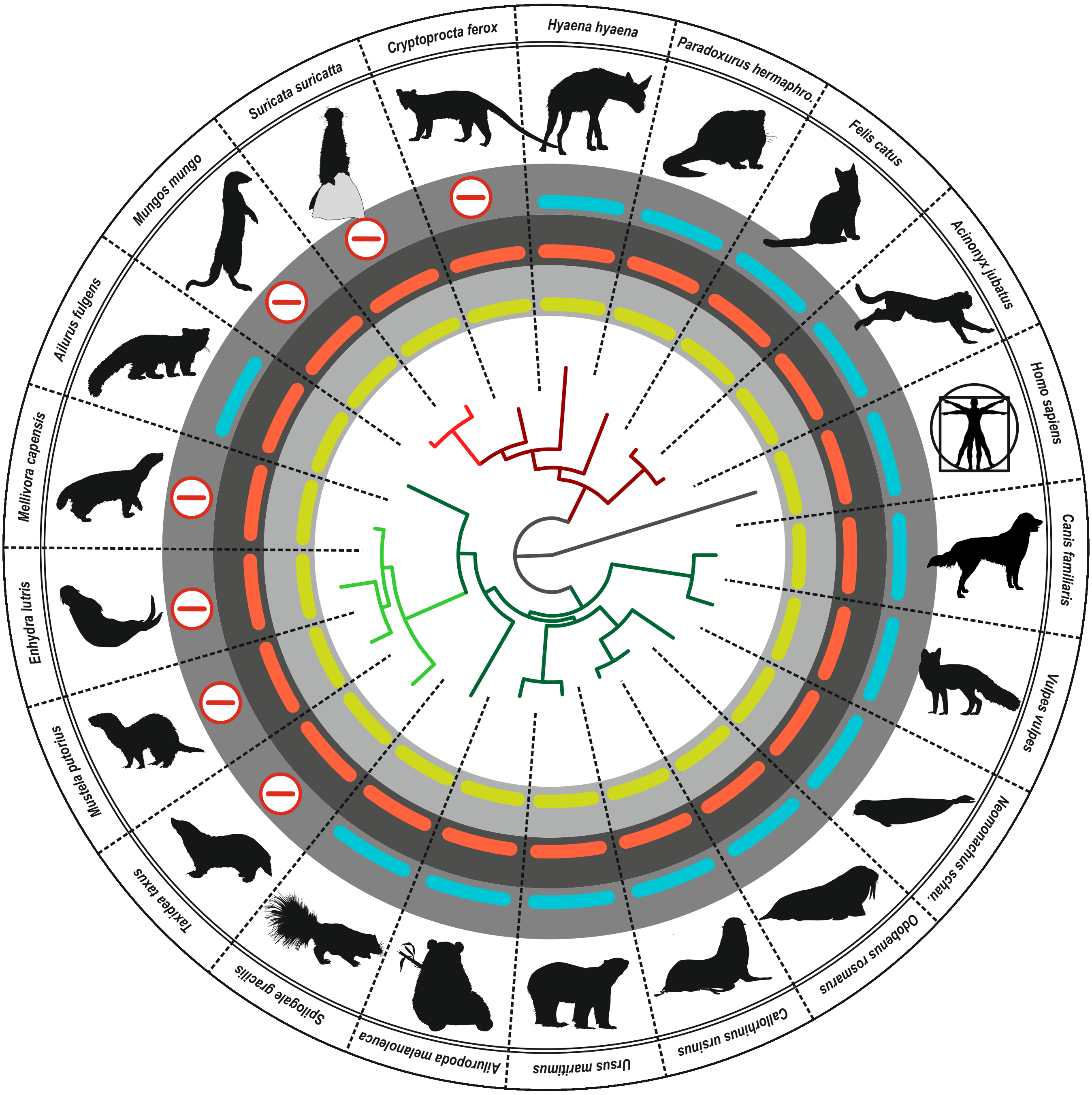

Hyaena hyaena
Paradoxurus hermaphro.
Felis catus
Acinonyx jubatus
Homo sapiens
Canis familiaris
Vulpes vulpes
Neomonachus schau.
Odobenus rosmarus
Callorhinus ursinus
Ursus maritimus
Ailuropoda melanoleuca
Spilogale gracilis
Taxidea taxus
Mustela putorius
Enhydra lutris
Mellivora capensis
Ailurus fulgens
Mungos mungo
Suricata suricatta
Cryptoprocta ferox

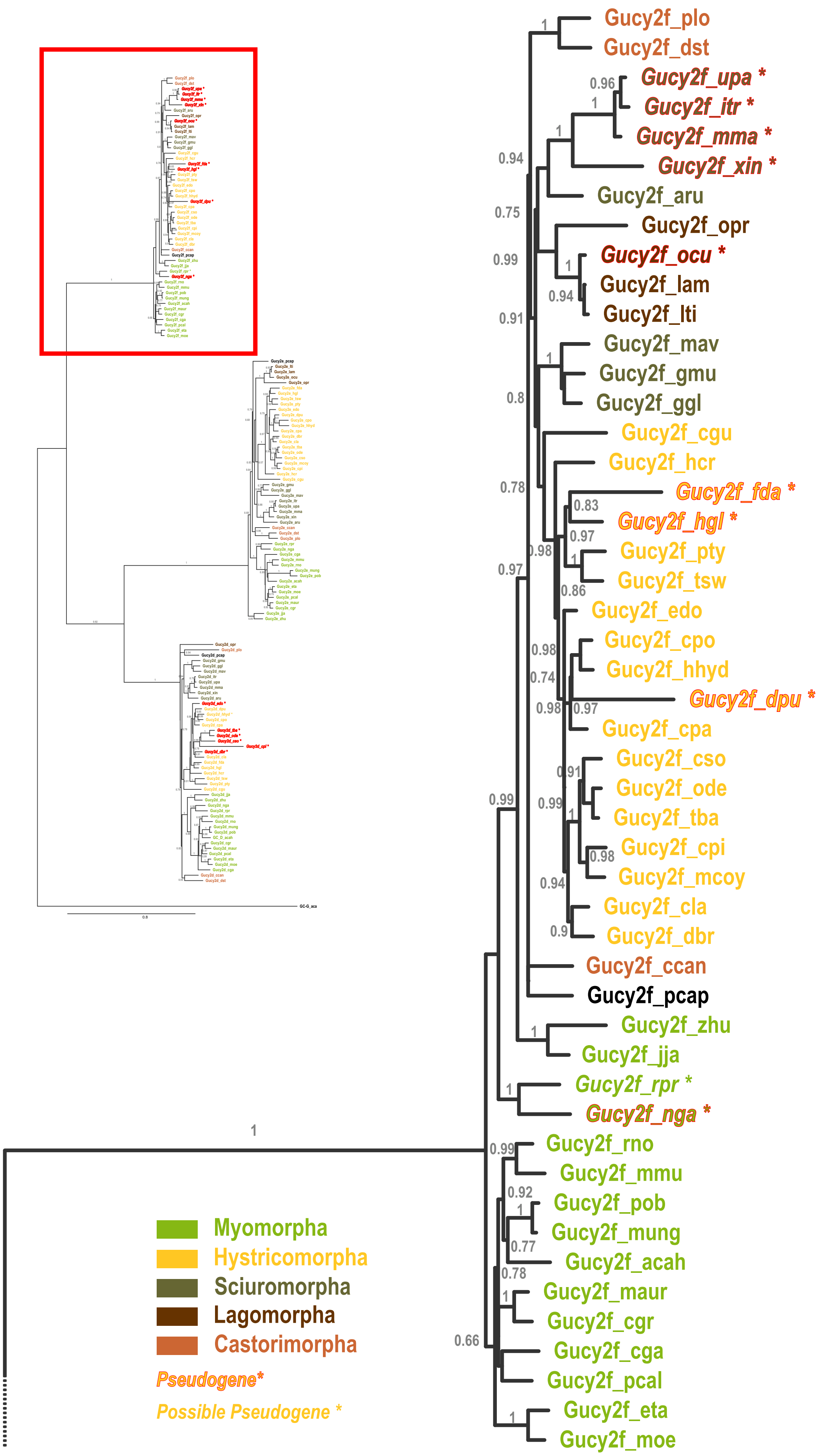

Gesemann & Neuhauss S11a

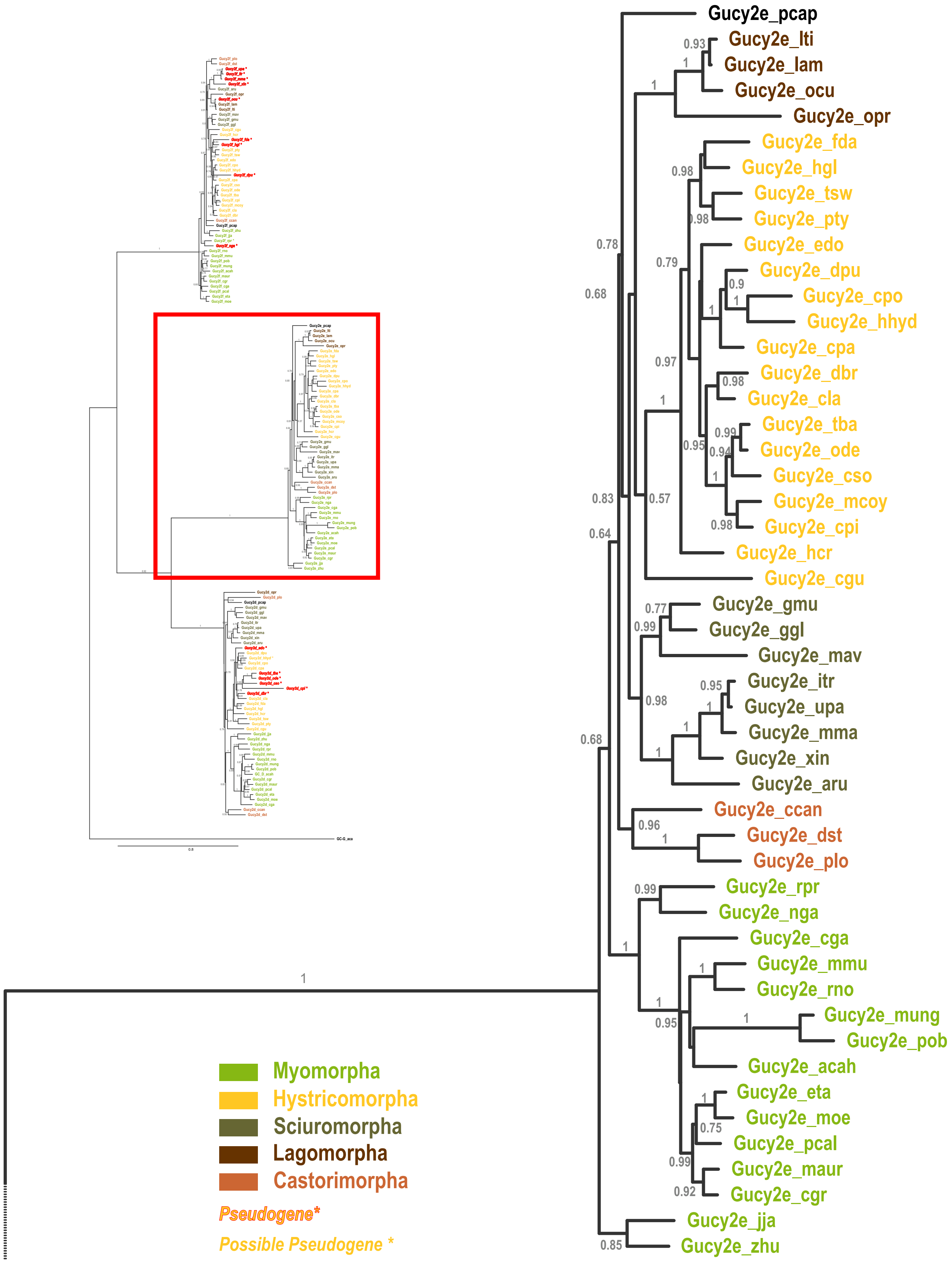

Gesemann & Neuhauss S11b

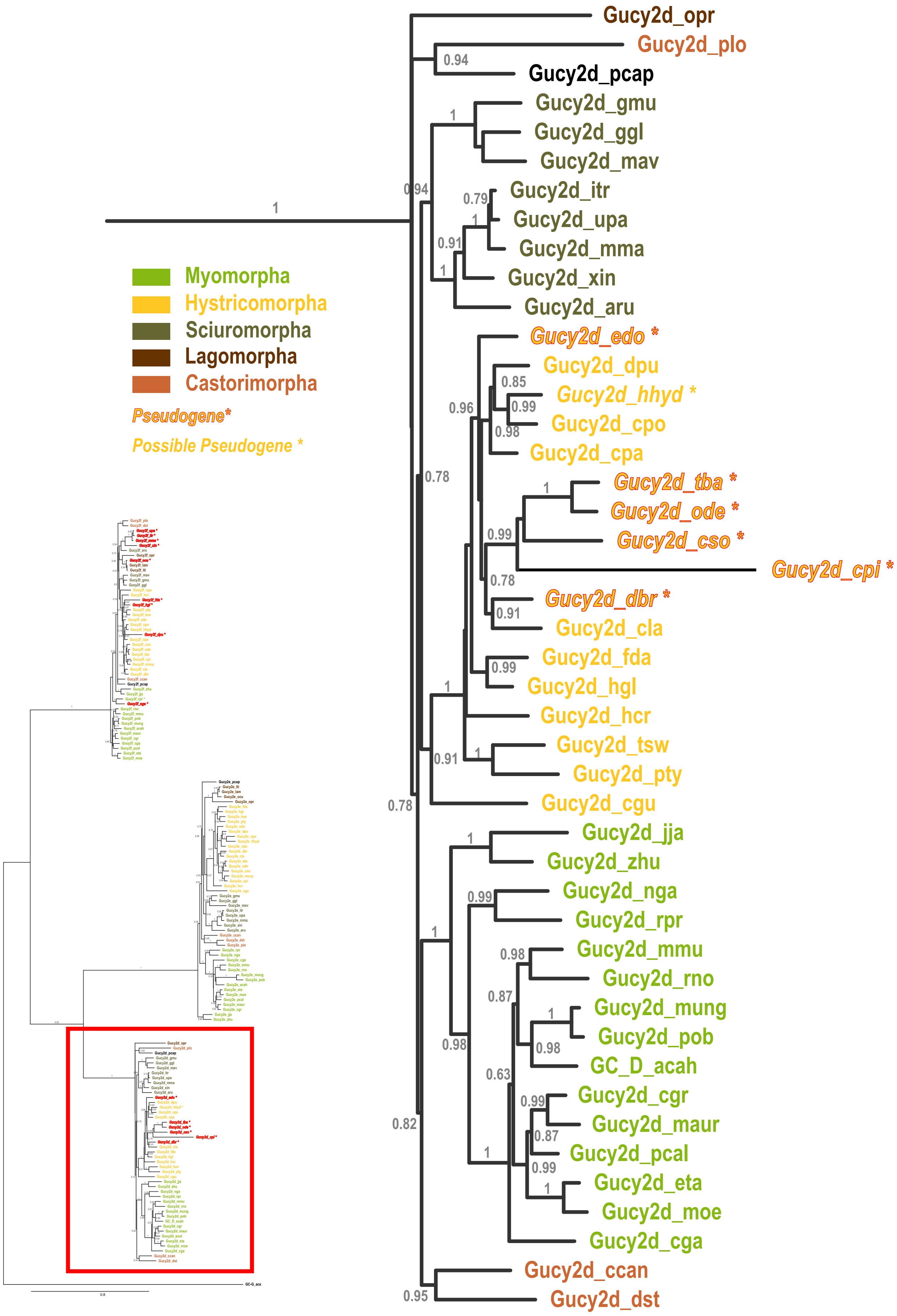

Gesemann & Neuhauss S11c

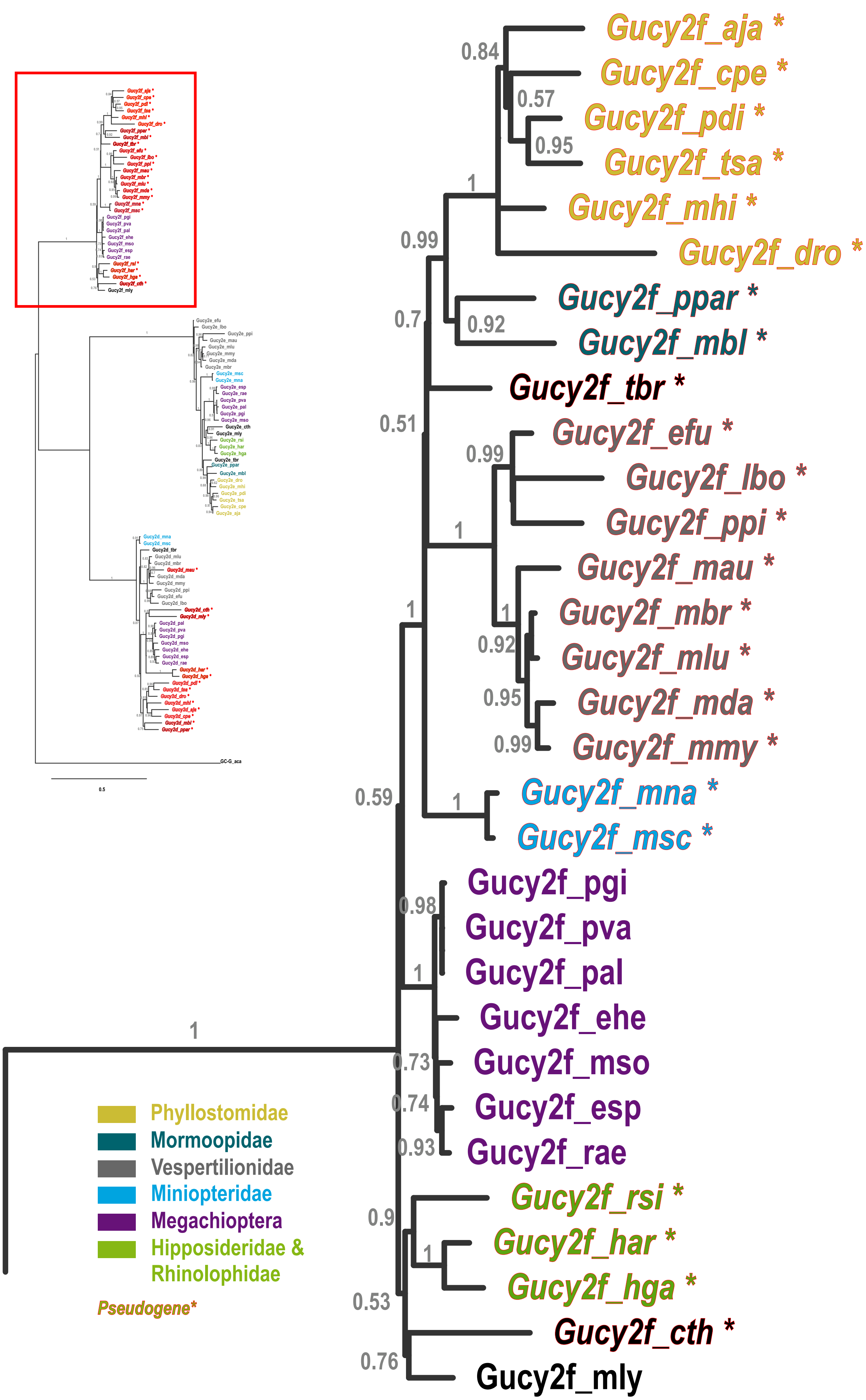

Gesemann & Neuhauss S12a

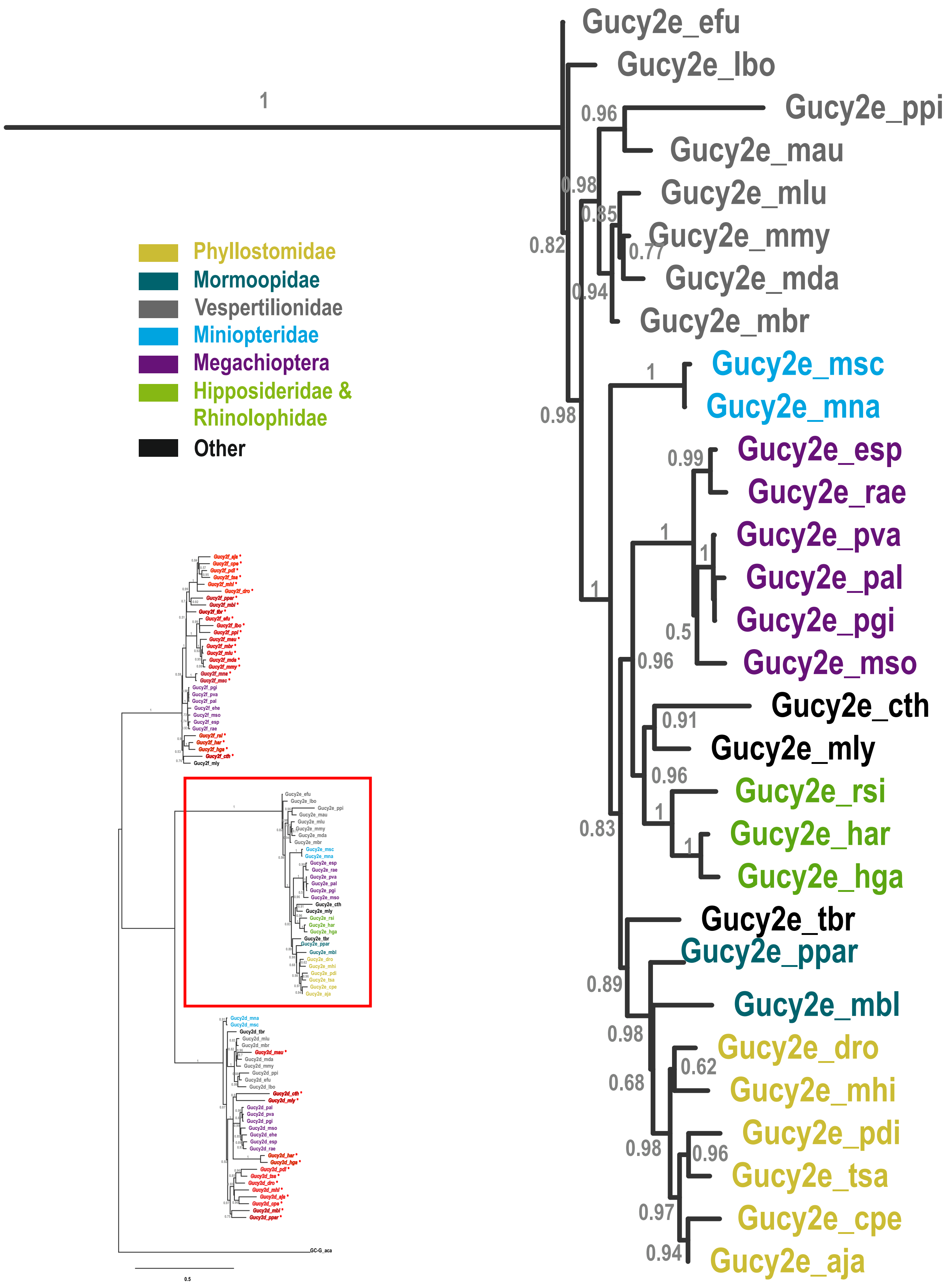

Gesemann & Neuhauss S12b

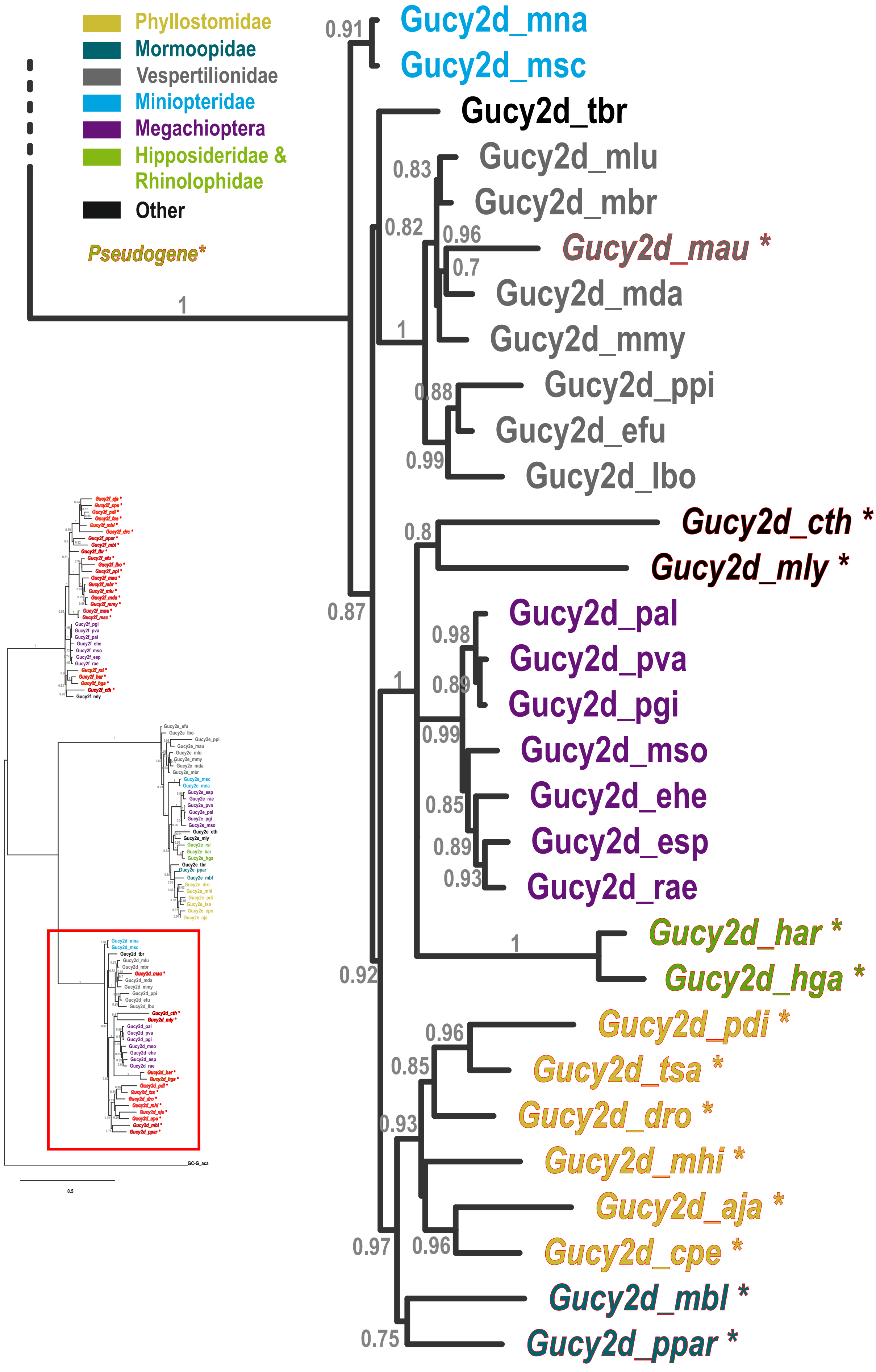

Gesemann & Neuhauss S12c
